# Ozone effects on blood biomarkers of systemic inflammation, oxidative stress, endothelial function, and thrombosis: The Multicenter Ozone Study in oldEr Subjects (MOSES)

**DOI:** 10.1371/journal.pone.0222601

**Published:** 2019-09-25

**Authors:** John R. Balmes, Mehrdad Arjomandi, Philip A. Bromberg, Maria G. Costantini, Nicholas Dagincourt, Milan J. Hazucha, Danielle Hollenbeck-Pringle, David Q. Rich, Paul Stark, Mark W. Frampton

**Affiliations:** 1 Department of Medicine, University of California at San Francisco, San Francisco, CA, United States of America; 2 Division of Environmental Health Sciences, School of Public Health, University of California, Berkeley, CA, United States of America; 3 San Francisco Veterans Affairs Medical Center, San Francisco, CA, United States of America; 4 Department of Medicine, University of North Carolina School of Medicine, Chapel Hill, NC, United States of America; 5 Center for Environmental Medicine, Asthma and Lung Biology, University of North Carolina, Chapel Hill, NC, United States of America; 6 Health Effects Institute, Boston, MA, United States of America; 7 New England Research Institute, Watertown, MA, United States of America; 8 Alpert Medical School, Brown University, Providence, RI, United States of America; 9 Department of Public Health Sciences, University of Rochester Medical Center, Rochester, NY, United States of America; 10 Department of Medicine, University of Rochester Medical Center, Rochester, NY, United States of America; 11 Department of Environmental Medicine, University of Rochester Medical Center, Rochester, NY, United States of America; Fondazione Toscana Gabriele Monasterio, ITALY

## Abstract

The evidence that exposure to ozone air pollution causes acute cardiovascular effects is mixed. We postulated that exposure to ambient levels of ozone would increase blood markers of systemic inflammation, prothrombotic state, oxidative stress, and vascular dysfunction in healthy older subjects, and that absence of the glutathione S-transferase Mu 1 (GSTM1) gene would confer increased susceptibility. This double-blind, randomized, crossover study of 87 healthy volunteers 55–70 years of age was conducted at three sites using a common protocol. Subjects were exposed for 3 h in random order to 0 parts per billion (ppb) (filtered air), 70 ppb, and 120 ppb ozone, alternating 15 min of moderate exercise and rest. Blood was obtained the day before, approximately 4 h after, and approximately 22 h after each exposure. Linear mixed effect and logistic regression models evaluated the impact of exposure to ozone on pre-specified primary and secondary outcomes. The definition of statistical significance was p<0.01. There were no effects of ozone on the three primary markers of systemic inflammation and a prothrombotic state: C-reactive protein, monocyte-platelet conjugates, and microparticle-associated tissue factor activity. However, among the secondary endpoints, endothelin-1, a potent vasoconstrictor, increased from pre- to post-exposure with ozone concentration (120 vs 0 ppb: 0.07 pg/mL, 95% confidence interval [CI] 0.01, 0.14; 70 vs 0 ppb: -0.03 pg/mL, CI -0.09, 0.04; p = 0.008). Nitrotyrosine, a marker of oxidative and nitrosative stress, decreased with increasing ozone concentrations, with marginal significance (120 vs 0 ppb: -41.5, CI -70.1, -12.8; 70 vs 0 ppb: -14.2, CI -42.7, 14.2; p = 0.017). GSTM1 status did not modify the effect of ozone exposure on any of the outcomes. These findings from healthy older adults fail to identify any mechanistic basis for the epidemiologically described cardiovascular effects of exposure to ozone. The findings, however, may not be applicable to adults with cardiovascular disease.

## Introduction

Exposure to air pollution is a well-established risk factor for cardiovascular morbidity and mortality. Most of the evidence supporting an association between air pollution and adverse cardiovascular effects involves exposure to particulate matter [1]. Tropospheric ozone, formed when nitrogen oxides and volatile organic compounds react in the lower atmosphere in the presence of sunlight, is known to have acute respiratory effects, with decrements in lung function and increases in lung inflammation, respiratory symptoms, and respiratory mortality and morbidity [2–4]. Recent epidemiological studies have also linked ozone exposure with increased cardiovascular mortality and morbidity, although the evidence is mixed. For example, Bell et al. [5] reported a 0.64% increase in the risk of cardiovascular and respiratory mortality (95% confidence interval [CI] 0.31%, 0.98%) associated with each 10 parts per billion (ppb) increase in the previous week’s ozone concentration. However, Bravo et al. [6] found no such acute association between increased ozone concentrations and cardiovascular mortality in the population of Sao Paulo, Brazil. Some investigators have reported increased risks of cardiovascular and/or cerebrovascular hospital and emergency room admissions associated with increased ozone concentrations in the days before the event [7–13], while others have not [14–22]. Most of these studies examined associations between health outcomes and multiple ambient air pollutants.

Meta-analyses have concluded that increased ambient ozone concentrations are associated with an increased risk/rate of stroke [23], but not myocardial infarction or heart failure [24, 25]. However, the ozone effect estimates from these meta-analyses are generally similar in magnitude, with statistical significance reflecting in part the number of studies used. Thus, taken together, the meta-analyses appear to support a very small increased risk of acute cardiovascular events associated with increased ozone concentrations in the days before the event. Indeed, the 2013 Integrated Science Assessment for ozone prepared by the US Environmental Protection Agency (EPA) [26], concluded there is likely a causal relationship between short-term exposure to ozone and both total and cardiovascular mortality.

Controlled exposure studies at pollutant concentrations relevant to ambient concentrations can explore the mechanistic basis for the associations observed in epidemiological studies between short-term exposures to ozone and acute cardiovascular disease outcomes, and identify markers of effect that would be useful in future observational (e.g., panel) studies. Recently, two controlled human exposure studies [27, 28] in healthy young adults, at ozone concentrations sufficient to elicit changes in pulmonary function (100 ppb for 4 h and 300 ppb for 2 h, respectively, with intermittent exercise), found effects on systemic inflammation. However, Frampton et al. [29] found no effects of 3-h exposures to 100 and 200 ppb ozone, with intermittent exercise, on measures of systemic and pulmonary vascular function, impedance cardiography, blood microparticles, or blood platelet activation. Thus, findings from controlled human exposure studies on the cardiovascular effects of ozone are inconsistent, and there are no studies focusing on effects in older subjects. Aging increases the risk of cardiovascular disease and may alter acute cardiovascular responses to inhaled ozone.

We postulated that initial reactions of inhaled ozone with lipid substrates on airways surfaces generate a variety of reactive chemicals that activate multiple airway cells. Mediators released from these cells enter the circulation and may cause activation of blood elements, notably platelets, and increase the production of acute phase reactants such as C-reactive protein (CRP). Activated endothelial and blood cells also generate and release membrane-enclosed vesicles (microparticles) that contain a variety of biologically active intracellular molecules. Such vesicles may serve an endocrine-like function by adhering to other cells and transferring their contents (e.g., microparticle-associated tissue factor). These systemic changes could result in prothrombotic as well as other cardiovascular effects.

Given that ozone exposure generates oxidative stress, individuals with impaired anti-oxidant defenses may be at increased risk for acute health effects. Soluble cytosolic GSH transferases (GST) belong to a supergene family with multiple classes which might be involved in detoxification of post-exposure inflammation-related oxidants or in the resolution of inflammation. The GST Mu 1 (GSTM1) gene (reviewed by Wu et al.) [30] is particularly noteworthy because about half the Caucasian population is genetically null for this enzyme. A controlled human exposure study has shown an increase in the late airway inflammatory response to ozone in GSTM1-null subjects [31, 32]. Because a prolonged inflammatory response would increase oxidative stress, it is plausible that the GSTM1-null state might contribute to post-ozone exposure cardiovascular changes. However, studies on the role of GSTM1 on susceptibility to cardiovascular effects are few. A recent controlled ozone exposure study [29] in young healthy volunteers did not support a role of GSTM1 in eliciting cardiac and vascular function effects.

The Multicenter Ozone Study in oldEr Subjects (MOSES) was a controlled human exposure study in which we exposed a relatively large number of healthy older adults, who may be more susceptible to air pollution-induced cardiovascular health effects than younger adults [33], to filtered air containing two levels of ozone (70 ppb and 120 ppb) and to filtered air without ozone. The study was designed to test the hypothesis that short-term exposure to ambient levels of ozone would induce acute cardiovascular responses through the following mechanisms: autonomic imbalance, systemic inflammation, and development of a pro-thrombotic vascular state. Secondary hypotheses were that ozone-induced acute cardiovascular responses would be associated with a) increased systemic oxidative stress and lung effects and b) the GSTM1 null genotype. We report here the results of MOSES with regard to blood biomarkers of systemic inflammation and a prothrombotic state. Secondary outcomes included markers of oxidative stress and endothelial function. Ozone effects on pulmonary and cardiovascular function in this study are reported elsewhere [34–36].

## Materials and methods

**Fig 1:**
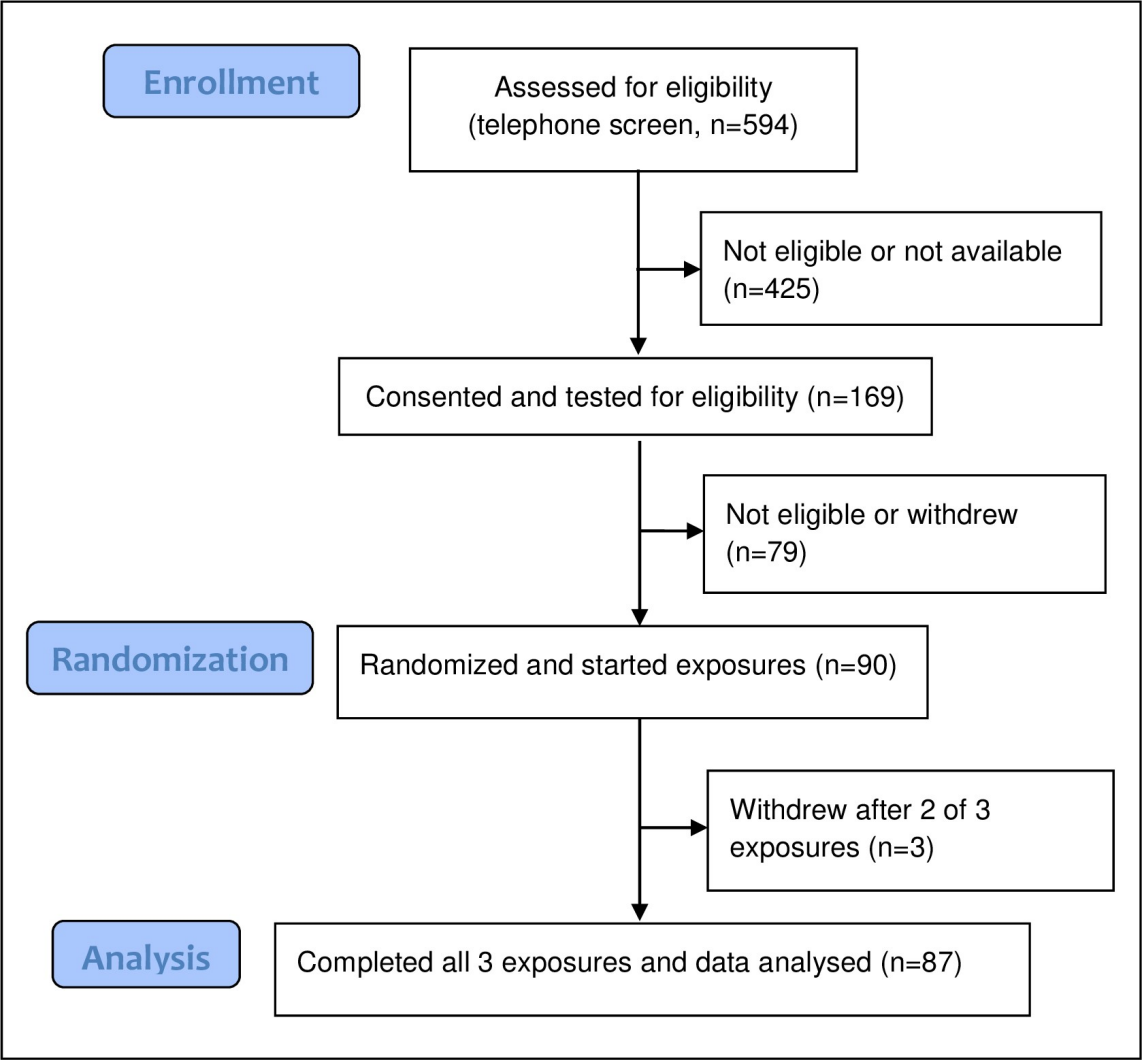
Participant flowchart- number of subjects at each stage of the study.

**Fig 2:**
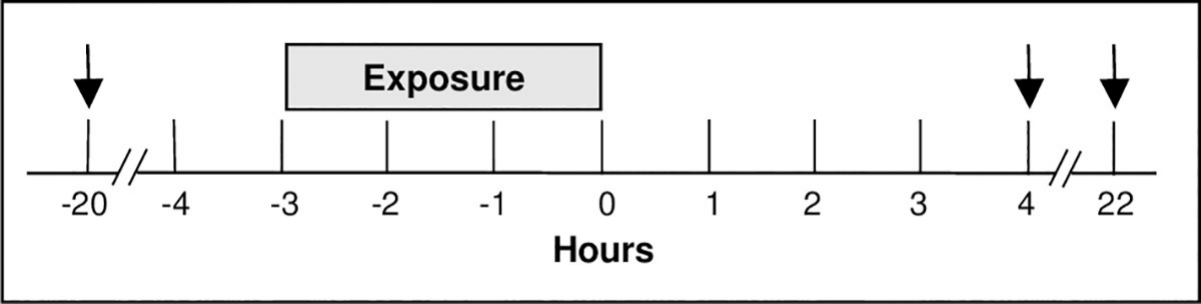
Timeline for each exposure session.

The design for MOSES has been previously described in detail [36]. The study protocol and other study documents are available at www.healtheffects.org. Briefly, MOSES was a controlled exposure study of the acute cardiovascular effects of ozone inhalation in healthy non-smoking adults (ages 55 to 70 years). The study was conducted in three clinical centers: the University of Rochester Medical Center (URMC), the University of North Carolina (UNC), and the University of California, San Francisco (UCSF); exposures were conducted from 2012 to 2015. The subjects underwent 3 randomized exposure sessions, each consisting of 3 consecutive days. At approximately noon on the first day of each session phlebotomy was performed; the second day involved randomized exposures to clean air (0 ppb), 70 ppb, and 120 ppb ozone for 3 h while alternately exercising and resting for 15 min, with phlebotomy 4 h after exposure; phlebotomy was performed again on the third day, 22 h after exposure. Exposure sessions were separated by a minimum of 2 weeks. Blood was collected from the antecubital vein of the arm not used for other procedures, into tubes containing sodium citrate. *In situ* platelet and endothelial activation were minimized by a short tourniquet time, minimizing trauma at needle entry, and discarding the first 5 mL of blood. Subjects with difficult venous access were excluded. The subjects, investigators, and study personnel, with the exception of the individual controlling the exposure chambers, were unaware of the nature of the exposures. The total duration of subject participation, from the screening visit to the final visit, typically varied between 3 and 6 months, but could be as long as 12 months. Fig 1 shows the number of subjects at each stage of the study, and Fig 2 shows the timeline for each exposure session.

### Subject recruitment

Written informed consent was obtained from all subjects at each clinical center. The study was approved by the institutional review board at each center and by the US EPA Human Subjects Research Review Official. A Certificate of Confidentiality was obtained from the US Department of Health and Human Services. The study was registered with ClinicalTrials.gov, Identifier NCT01487005.

Subjects were recruited through advertisements at the three clinical centers. Inclusion criteria were as follows: nonsmoking males and females of all ethnic backgrounds, ≥55 and ≤70 years of age; normal spirometry (forced expiratory volume in 1 s (FEV_1_) and forced vital capacity (FVC) ≥75% of NHANES predicted values and FEV_1_/FVC ≥0.65); ability to complete the training exercise regimen chosen to induce an inspired ventilation rate of 15–17 L body temperature and pressure, saturated (BTPS) /min/m^2^ body surface area (BSA), without exceeding 80% of predicted maximal heart rate; normal baseline 12-lead resting ECG, and absence of significant ST depression while performing the 15-min required level of exercise targeted for the exposure period; ability to avoid a specific list of medications and supplements.

Exclusion criteria were as follows: non-English speaking; reported chronic cardiovascular or respiratory disease, diabetes, or other organ or system dysfunction; active psychiatric disorders that would interfere with the subject’s ability to understand and participate in the study; current drug or alcohol abuse; ever-smokers (smoked tobacco or marijuana during the last five years, or with history of >10 pack-years for tobacco or >1 joint-year for marijuana, or living with a smoker who smokes inside the house); plasma cotinine level >3 ng/mL; body mass index (BMI) >35 and <18; blood pressure >140 systolic or >90 diastolic or on anti-hypertension medications other than diuretics; pregnancy or nursing (breastfeeding); on the following medications: prednisone, statins, beta-blockers, anticoagulants, current systemic estrogen therapy, tamoxifen; and current occupational exposures to high levels of vapors, dust, gases, or fumes.

Subjects were not studied within 6 weeks of a respiratory infection and were asked to abstain from caffeinated and alcoholic beverages starting with lunch on the day before the exposure through the post-exposure day.

### Exposure generation and characterization

The exposures took place in ventilated, climate-controlled chambers at each of the clinical centers. The chamber characteristics and specific protocols for generating and measuring ozone were site-specific and are described elsewhere [36].

All three centers generated ozone by silent electric-arc discharge in breathing-quality (USP grade) oxygen from a gas cylinder. The dilution air entering the exposure chamber was purified by passing it through Purafil, charcoal, and HEPA filters. The effluent from the ozone generator was mixed with the filtered air immediately before entering the chamber. The ozone output was controlled by varying the voltage of the generator and adjusting the flow rate. Temperature and relative humidity were targeted at 22°C and 40%, respectively. The concentration of ozone breathed by the subject was monitored continuously during the exposure by calibrated and externally certified analyzers.

### Outcomes

The MOSES outcomes were selected based on hypothesized cardiovascular mechanisms of action of ozone. For each potential mechanistic pathway we identified one or more primary outcomes (listed in **bold** below) and several secondary outcomes, based on either previous evidence in the ozone health effects literature or clinical relevance.

In this paper, we report effects on peripheral blood biomarkers of systemic inflammation, oxidative stress, and endothelial function (**CRP**, interleukin-6 [IL-6], 8-isoprostane, P-selectin, nitrotyrosine, endothelin-1 [ET-1]), and a prothrombotic state (**microparticle-associated tissue factor activity [MP-TFA]**, von Willebrand factor (vWF), fibrinogen, and flow-cytometric indices of platelet activation, including **monocyte-platelet conjugate count**, activated platelet [CD62P^+^] count, platelet-derived microparticle [CD42b^+^] count, activated platelet-derived microparticle [CD42b^+^/62P^+^] count, tissue factor expressing microparticle [CD142^+^] count, and CD40 ligand-expressing microparticle [CD154^+^] count).

### Assays

#### Microparticle-associated tissue factor activity

Plasma samples for the MP-TFA assay were analyzed as previously described [37, 38], at the MP-TFA Core Laboratory at UNC. Briefly, microparticles were isolated from plasma by adding 1 mL calcium-free HEPES buffer to each 200 uL sample and pelleting at 20,000 g for 15 min at 4°C. The pellet was washed and resuspended in the same buffer. The MP-TF assay depends on adding calcium in the presence of activated human factor VII (FVIIa) (Enzyme Research Lab). The TF-FVIIa complex plus Ca^++^ activates added factor X (FX) (Enzyme Research Lab). The resulting FXa was then assayed colorimetrically (absorbance at 405 nm) in the MP pellet suspension along with suitable controls and standards in a 96-well plate with Pefachrome FXa 8595 (Pentapharm #085–27, Aesch, Switzerland) [37]. An essential control, the determination of TF-independent FXa generation, in this system involved first neutralizing TF with anti-human TF antibody (HTF-1) (Becton-Dickinson Biosciences #550252, San Jose,CA). Two plasma samples, in duplicate, were analyzed for each subject time point. Data are reported as the average of the two measurements.

#### Platelet activation and circulating microparticles

We measured blood platelet activation and circulating microparticles using immunofluorescence and flow cytometry (FC), with modifications of methods previously reported [39]. The FC analyses were conducted at each clinical center within 1 hour of the blood draw, on 18-color LSRII flow cytometers (Becton Dickinson Biosciences, San Jose, CA), using the following laser options: blue 488, 515/20 nm band pass; red 633, 620/20 nm band pass; and green 532, 575/25 nm band pass. Standardized beads were run simultaneously with the subject samples for sizing (SORP-NIST sizing beads, obtained by special order from Becton Dickinson), event counting (AccuCount Beads, ACBP-20-10, obtained by special order from Spherotech, Lake Forest, IL), and fluorescence intensity (Rainbow Beads, RCP-60-5, Spherotech). Platelets and microparticles were characterized both unstimulated and “primed” with thrombin receptor activator peptide (TRAP)-6 (H-8365, Bachem Americas Inc., Torrance, CA), at a concentration (2.34 μM) just below that causing detectable activation using our methods. Marker ligand measurements included both counts of positive events and mean fluorescence intensity of the event population. Data were collected in list mode and sent to the Flow Core at URMC for analysis when each subject’s exposures were completed.

The list mode data collected at each Center were analyzed using FloJo software (TreeStar, Ashland, OR). Platelets and monocyte-platelet conjugates were analyzed using a method adapted from Li et al. [40]. Microparticles were identified using the sizing beads and surface markers of the cells of origin [41]. Before starting the study, the analysis protocols and procedures were developed and validated using a series of pilot blood samples from healthy volunteers at each study site, with analysis at the Flow Core, to assure consistent results among the study sites. All data were analyzed at the Flow Core by the same individual. A second technician performed duplicate analyses using the same methods on complete data from three subjects, one from each site, to confirm that results were not operator-dependent.

#### Commercial laboratory measurements

CRP, IL-6, 8-isoprostane, nitrotyrosine, fibrinogen, ET-1, and vWF were measured in blood plasma by the AssayGate commercial laboratory (Ijamsville, MD). The specific assays used are described below. The laboratory utilized custom-designed antibody kits that are proprietary. The limit of detection (LOD) of each assay was defined as the value calculated from the standard curve at the point lying 2 standard deviations above the mean background media fluorescence intensity for 10 replicates.

Luminex bead-based multiplex immunoassays were used to measure CRP, fibrinogen, vWF, IL-6, and P-selectin (Luminex® xMAP® technology (Austin, TX). Dilution was performed for samples falling outside the range of the standard curves. Samples were tested in duplicate. Positive controls with known analyte concentrations and negative controls on each bead plate allowed for assay quality assurance. Sandwich enzyme-linked immunosorbent assays (ELISA) were used to measure ET-1 and nitrotyrosine. A competitive ELISA was used to measure 8-isoprostane. A 4-parameter standard curve was prepared relating color intensity to the concentration of the target on each plate. Positive and negative controls on each plate allowed for assay quality assurance.

### Statistical anaysis

Power calculations for selected outcomes were performed prior to study initiation and again during the study. Details of these analyses can be found at https://www.healtheffects.org/system/files/MOSES-Pt1-AddtlMaterials-5.pdf.

Outcomes were calculated by subtracting the pre-exposure value from each post-exposure value. Any of these outcome variables that were not normally distributed, via the Kolmogorov-Smirnov test, were transformed using the natural logarithm (adding 0.001 to 0 values). This resulted in more normal distributions of these outcomes. Outcome variables that were normally distributed were summarized using means with SDs, and those that were skewed, and transformed as described above, were summarized using both means with SDs and medians with inter-quartile ranges. Age was treated as a continuous value and centered by subtracting the mean age for the cohort.

Linear mixed effect models, with a random subject effect and a ‘variance components’ covariance structure, were used to evaluate the impact of exposure to ozone on the pre-specified primary and secondary outcomes. Ozone was treated as a categorical (3-level) variable, with clinical center and time of measurement (also categorical) included in all models. When there was a statistically significant effect of ozone on an outcome, the ozone by time interaction was then assessed to determine whether the ozone effect was consistent across the individual post-exposure times. Since this study involved a large number of comparisons, we pre-specified a limited number of outcomes as primary (three of these primary variables are reported here), and used α = 0.01 as the threshold for statistical significance, with α = 0.05 as the threshold for marginal significance. Results were then interpreted within the context of coherence among related variables, and plausibility. Significant changes in secondary variables were to be considered hypothesis-generating but not definitive, unless there were changes consistent with effects on the primary outcome variable(s) for that response pathway. In that case, the secondary variable was considered supportive of the findings for the primary variable(s). Analyses were performed using SAS, Version 9.3 or later (SAS Institute, Cary, NC) and R version 3.2.2 or later (The R Foundation for Statistical Computing).

## Results

A total of 1,062 individuals responded to the advertisements of the three clinical centers, and 594 of these underwent telephone screening (Fig 1). Eighty-seven subjects completed all three exposures and were included in the analyses; their characteristics are shown in Table 1. Fifty-two (60%) were women and 35 were men. The mean (standard deviation [SD]) age of the subjects was 59.9 (4.5) years. The majority of subjects (88%) identified themselves as white, with African American being the next most common with 5 (6%). Overall, 50 (57%) of the subjects were GSTM1 null, and the prevalence was consistent across the three centers.

**Table 1:** Characteristics of MOSES subjects by center.

	URMC (N = 32) (%)	UNC (N = 29) (%)	UCSF (N = 26) (%)	Overall (N = 87) (%)
**Gender**				
Male	12 (37.5)	9 (31.0)	14 (53.8)	35 (40.2)
Female	20 (62.5)	20 (69.0)	12 (46.2)	52 (59.8)
**Race**				
American Indian	1 (3.1)	0 (0)	0 (0)	1 (1.1)
Asian	0 (0)	0 (0)	2 (7.7)	2 (2.3)
Black	1 (3.1)	4 (13.8)	0 (0)	5 (5.7)
White	28 (87.5)	25 (86.2)	23 (88.5)	76 (87.4)
Hawaiian	0 (0)	0 (0)	1 (3.8)	1 (1.1)
Unknown	1 (3.1)	0 (0)	0 (0)	1 (1.1)
**GSTM1**				
Wild type	15 (46.9)	13 (44.8)	9 (34.6)	37 (42.5)
Null	17 (53.1)	16 (55.2)	17 (65.4)	50 (57.4)
**Age (yrs)**	59.1 ±3.8^a^	60.4 ±5.1	60.3 ±4.7	59.9 ±4.5
**BMI (kg/m**^**2**^**)**	25.0 ±2.4	24.8 ±3.7	24.8 ±3.6	24.9 ±3.2
**Systolic BP (mmHg)**	122.4 ±11.4	120.4 ±9.7	122.2 ±12.8	121.7 ±11.2
**Diastolic BP (mmHg)**	69.0 ±7.5	76.1 ±7.8	73.7 ±10.7	72.8 ±9.1
**Cholesterol Total(mg/dL)**	208.3 ±34.7	215.3 ±30.7	215.8 ±47.5	212.9 ±37.6
**LDL Calc (mg/dL)**	118.4 ±30.0	119.6 ±29.2	123.7 ±41.8	120.4 ±33.4
**% predicted FEV**_**1**_	104.0 ±12.8	102.4 ±13.9	102.6 ±12.9	103.0 ±13.1

URMC, University of Rochester Medical Center; UNC, University of North Carolina; UCSF, University of California San Francisco; GSTM1, glutathione S-transferase Mu 1 gene; BMI, body mass index; BP, blood pressure; LDL, low-density lipoprotein cholesterol; FEV_1_, forced expiratory volume in 1 s.

^a^Mean±SD.

### Exposure conditions

The ozone concentrations in the chambers of all three centers were close to the target values. The mean (SD) temperature was 22.3 (0.7)°C, compared to the target of 22°C, and the mean (SD) relative humidity was 41.4 (3.0) %, compared to the target of 40%.

### Effects of ozone on blood biomarkers

#### Systemic inflammation, oxidative stress, and endothelial function

Ozone caused no change in plasma CRP, IL-6, 8-isoprostane, or P-selectin. Descriptive statistics are shown in Table 2; the distribution of values for CRP is skewed (Table 3). Linear regression analysis results are shown in Table 4. There were no statistically significant associations of ozone with any of these biomarkers, and no interactions with sex, age, or GSTM1 status (Tables A-X in S1 Appendix). In a sensitivity analysis, there were no significant ozone effects on CRP when subjects with baseline CRP values above the median were excluded.

**Table 2:** Descriptive statistics for systemic inflammatory, oxidative stress, and endothelial function outcomes.

Outcome^a^	0 ppb			70 ppb			120 ppb		
	N	Mean	SD	N	Mean	SD	N	Mean	SD
**CRP** (mg/L)									
Pre Exposure	85	2.72	3.56	85	2.94	3.66	84	3.05	4.01
4 h Post Exposure	85	2.49	3.42	85	2.48	3.43	84	2.71	3.67
22 h Post Exposure	82	2.75	3.66	85	2.81	3.83	82	2.70	3.84
IL-6 (pg/mL)									
Pre Exposure	85	3.25	3.14	85	3.26	3.03	84	3.03	2.65
4 h Post Exposure	85	3.28	2.85	85	2.84	2.21	84	2.83	2.34
22 h Post Exposure	82	3.34	2.80	85	3.31	3.33	82	2.89	2.73
8-Isoprostane (pg/mL)									
Pre Exposure	85	61.65	30.12	85	61.07	31.49	84	61.99	33.72
4 h Post Exposure	85	57.98	27.13	85	55.38	25.11	84	59.55	30.86
22 h Post Exposure	82	63.00	32.74	85	59.89	28.69	82	60.40	26.29
P-selectin (ng/mL)									
Pre Exposure	85	68.59	76.95	85	69.93	69.88	84	65.43	55.57
4 h Post Exposure	85	63.09	48.74	85	55.62	35.02	84	62.10	41.30
22 h Post Exposure	82	116.71	285.96	85	77.06	85.65	82	84.26	141.08
Nitrotyrosine (nM)									
Pre Exposure	85	621.6	885.8	85	641.0	1066.7	84	606.5	807.2
4 h Post Exposure	85	629.3	905.2	85	659.1	1169.0	84	592.9	758.6
22 h Post Exposure	82	678.0	1010.3	85	647.6	1138.5	82	514.9	437.0
ET-1 (pg/mL)									
Pre Exposure	85	1.27	0.42	85	1.26	0.35	84	1.18	0.40
4 h Post Exposure	85	1.24	0.43	85	1.22	0.36	84	1.24	0.47
22 h Post Exposure	82	1.23	0.51	85	1.17	0.40	82	1.20	0.44
Fibrinogen (μg/mL)									
Pre Exposure	85	1649.4	2270.0	85	1790.0	2118.4	84	1319.0	1304.7
4 h Post Exposure	85	1459.2	1836.7	85	1520.8	1625.4	84	1346.0	1321.3
22 h Post Exposure	82	1657.1	2069.0	85	1544.9	1795.9	82	1749.0	1864.5

CRP, c-reactive protein; IL-6, interleukin-6; ET-1, endothelin-1.

^a^Primary outcome bolded.

**Table 3:** Median and IQR of skewed data for CRP.

CRP (mg/L)	0 ppb			70 ppb			120 ppb		
	N	Median	IQR	N	Median	IQR	N	Median	IQR
Pre Exposure	85	1.51	(0.59, 3.43)	85	1.91	(0.65, 3.75)	84	1.73	(0.65, 3.17)
4 h Post Exposure	85	1.48	(0.65, 2.97)	85	1.44	(0.52, 2.87)	84	1.33	(0.67, 2.88)
22 h Post Exposure	82	1.39	(0.60, 3.60)	85	1.40	(0.63, 3.27)	82	1.45	(0.72, 3.61)

CRP, c-reactive protein.

**Table 4:** Main analysis: Ozone effects on systemic inflammatory, oxidative stress, and endothelial function outcomes^a^.

Outcome^b^	Ozone (ppb)	Difference in estimates^c^	95% CI	Type III SS P-value
**CRP** (mg/L)	120	-0.15	-0.54, 0.23	0.655
	70	-0.16	-0.54, 0.23	
	0	---	---	
IL-6 (pg/mL)	120	-0.22	-0.73, 0.29	0.567
	70	-0.25	-0.75, 0.26	
	0	---	---	
8-isoprostane (pg/mL)	120	-0.88	-5.87, 4.10	0.749
	70	-1.91	-6.85, 3.04	
	0	---	---	
P-selectin (ng/mL)	120	-14.06	-42.37, 14.26	0.235
	70	-24.28	-52.41, 3.85	
	0	---	---	
Nitrotyrosine (nM)	120	-41.5	-70.1, -12.8	0.017
	70	-14.2	-42.7, 14.2	
	0	---	---	
ET-1 (pg/mL)	120	0.07	0.01, 0.14	0.008
	70	-0.03	-0.09, 0.04	
	0	---	---	

SS, sum of squares; CRP, c-reactive protein; IL-6, interleukin-6; ET-1, endothelin-1.

^a^Linear mixed effect models (see Methods).

^b^Primary outcome bolded.

^c^Change from pre- to post-exposure for 120 ppb compared to 0 ppb ozone, and 70 ppb compared to 0 ppb ozone.

**Fig 3:**
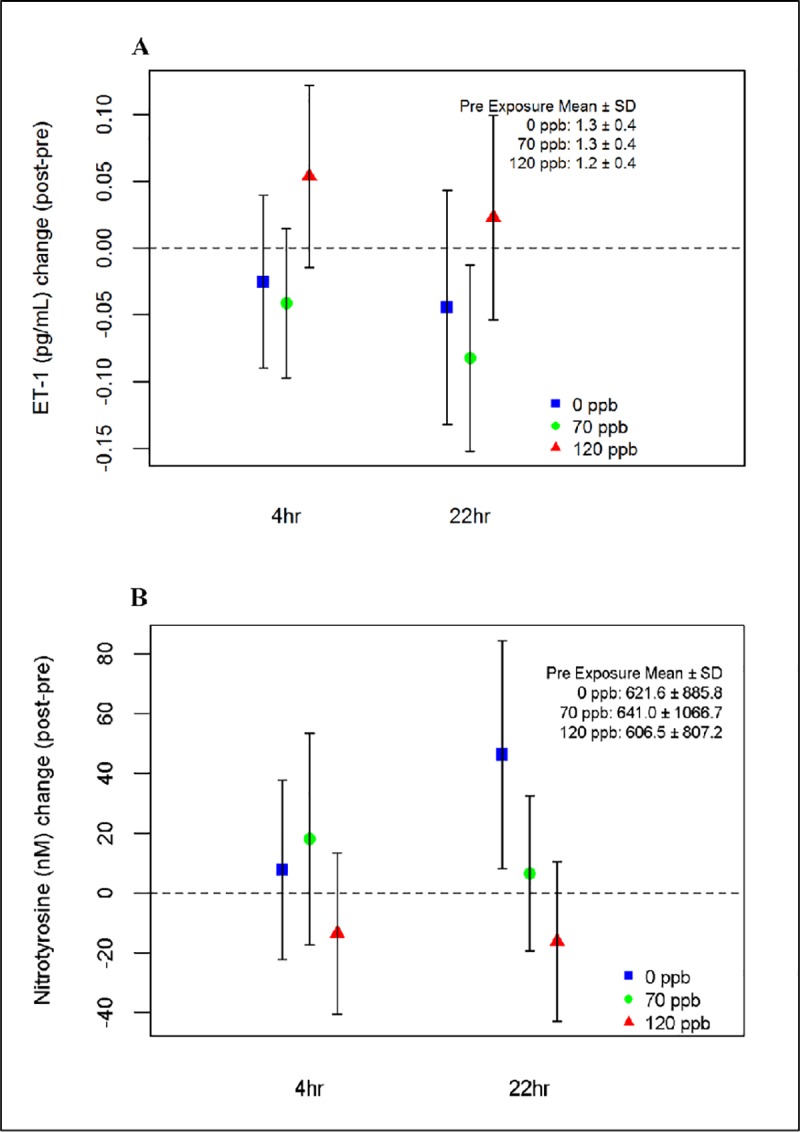
Effect of ozone on nitrotyrosine and ET-1. Changes from pre-exposure in (A) ET-1 and (B) nitrotyrosine for exposures to 0, 70, and 120 ppb ozone at 4 h and 22 h post-exposure. Pre-exposure mean and standard deviation (SD) values are shown as an insert. The whiskers represent 95% confidence intervals.

Nitrotyrosine decreased after 120 ppb ozone. In linear regression analysis, ozone effects on nitrotyrosine were marginally statistically significant (p = 0.017). As shown in Fig 3, panel B, nitrotyrosine increased 22 hr after 0 ppb ozone exposure but decreased in a concentration-response pattern after 70 and 120 ppb compared to 0 ppb. Nitrotyrosine was unchanged at 4 hr. We found no significant interaction of ozone with sex, age, or GSTM1 status (Tables Y-Ff in S1 Appendix).

ET-1 increased after 120 ppb, but not after 70 ppb ozone. In linear regression analysis, ozone effects on ET-1 were statistically significant (p = 0.008, Table 4). While there was no change in ET-1 after 70 ppb compared to 0 ppb at either 4 h or 22 h after exposure, it increased at both time points after 120 ppb compared to 0 ppb (Fig 3, panel A). We found no statistically significant interactions of ozone with sex, age, or GSTM1 genotype (Tables Gg-Ll in S1 Appendix).

#### Prothrombotic vascular status

Ozone exposure did not increase platelet activation. The summary statistics for each primary and secondary marker are shown in Table 5, and the model results are shown in Table 6. There were no significant ozone effects on any of the markers of platelet activation. We found a marginally significant interaction between ozone and age for monocyte-platelet conjugates (p = 0.013, Tables Oo and Pp in S1 Appendix). For every 1-year increase in age, exposure to 70 ppb ozone resulted in a 2.2 lower monocyte-platelet conjugate count (95% CI, -0.7, -3.6), and exposure to 120 ppb ozone resulted in a 1.5 lower monocyte-platelet conjugate count (95% CI, -0.06, -3.0; p = 0.041). There was no significant interaction with sex or GSTM1 (Tables Qq-Tt in S1 Appendix).

**Table 5:** Descriptive statistics for prothrombotic vascular outcomes.

Outcome^a^	0 ppb			70 ppb			120 ppb		
	N	Mean	SD	N	Mean	SD	N	Mean	SD
**Monocyte-platelet** **conjugate count**									
Pre Exposure	78	50.1	47.7	73	47.4	42.0	76	52.9	49.7
4 h Post Exposure	78	51.2	42.6	73	44.8	31.2	76	51.0	42.2
22 h Post Exposure	74	45.1	40.1	73	42.7	27.8	74	49.7	41.9
Activated platelet count									
Pre Exposure	82	20927.7	21222.8	80	18031.1	18312.0	82	19654.0	16465.4
4 h Post Exposure	81	17532.7	12192.9	79	16117.8	14006.0	82	16152.5	13679.8
22 h Post Exposure	78	20587.0	37302.4	79	15922.9	14867.4	80	16487.2	13555.2
**MP-TFA** (pg/mL)									
Pre Exposure	84	0.149	0.170	84	0.130	0.178	86	0.154	0.201
4 h Post Exposure	84	0.147	0.192	84	0.136	0.154	86	0.177	0.199
22 h Post Exposure	80	0.143	0.150	84	0.121	0.141	84	0.152	0.159
Platelet MP count									
Pre Exposure	81	5205.2	4028.4	80	5101.1	2940.9	81	5397.6	4112.5
4 h Post Exposure	80	4677.8	3207.6	79	5025.9	3619.3	81	5038.3	3314.0
22 h Post Exposure	77	4236.3	2237.8	79	4557.0	2554.8	79	4948.2	3251.6
Activated platelet MP count									
Pre Exposure	81	832.3	1076.6	80	723.0	752.1	81	763.0	561.3
4 h Post Exposure	80	686.8	620.1	79	706.8	679.5	81	685.6	448.8
22 h Post Exposure	77	590.8	415.8	79	603.3	427.4	79	668.1	422.4
CD142+MP count									
Pre Exposure	80	25033.5	40847.4	80	22555.8	32787.7	80	29834.9	56386.7
4 h Post Exposure	79	22881.8	50998.0	79	15907.7	20139.5	80	20071.2	31808.0
22 h Post Exposure	76	15082.1	23611.4	79	17851.5	31657.5	78	19492.0	28543.7
CD40 Ligand+ MP count									
Pre Exposure	80	33283.4	57668.5	80	32007.5	48664.5	80	37502.8	66018.2
4 h Post Exposure	79	30629.2	60380.1	79	23561.1	31098.3	80	24390.1	31387.4
22 h Post Exposure	76	21909.8	30699.2	79	20977.3	23635.2	78	26681.8	36682.2
Platelet count (1000/uL)									
Pre Exposure	84	236.8	51.8	85	237.2	59.3	85	233.9	51.3
4 h Post Exposure	83	231.4	48.5	85	230.7	61.2	84	228.8	52.3
22 h Post Exposure	80	230.3	54.9	85	226.7	61.5	81	225.9	52.7
vWF (ng/mL)									
Pre Exposure	85	23774.0	24127.0	85	22101.0	26088.1	84	22450.3	21076.3
4 h Post Exposure	85	23606.5	26519.2	85	23198.3	26646.7	84	21234.9	19271.9
22 h Post Exposure	82	24703.3	25631.4	85	22359.2	36850.5	82	21645.4	24122.8
Fibrinogen (μg/mL)									
Pre Exposure	85	1649.4	2270.0	85	1790.0	2118.4	84	1319.0	1304.7
4 h Post Exposure	85	1459.2	1836.7	85	1520.8	1625.4	84	1346.0	1321.3
22 h Post Exposure	82	1657.1	2069.0	85	1544.9	1795.9	82	1749.0	1864.5

MP-TFA, microparticle-associated tissue factor activity; MP, microparticles; vWF, von Willebrand factor.

^a^Primary outcomes are bolded.

**Table 6:** Main analysis: Ozone effects on prothrombotic vascular outcomes.

Outcome^a^	Ozone (ppb)	Estimates^b^	95% CI	Type III SS P-value
**Monocyte-platelet** **conjugates** (count)	120	-0.2	-6.8, 6.4	0.873
	70	-1.6	-8.3, 5.0	
	0	---	---	
Activated platelets (count)	120	-1437.3	-5686.6, 2812.0	0.781
	70	-314.3	-4591.6, 3962.1	
	0	---	---	
**MP-TFA** (pg/mL)	120	0.009	-0.030, 0.048	0.772
	70	-0.005	-0.044, 0.034	
	0	---	---	
Platelet MP (count)	120	213.7	-382.6 810.0	0.524
	70	341.7	-256.9, 940.3	
	0	---	---	
Activated platelet MP (count)	120	75.3	-92.6, 243.2	0.514
	70	92.9	-75.7, 261.5	
	0	---	---	
CD142+ MP (count)	120	-4444.2	-12932.0, 4043.6	0.551
	70	-927.1	-9418.6, 7564.3	
	0	---	---	
CD40L MP (count)	120	-6516.9	-15307.0, 2273.6	0.306
	70	-5186.7	-13984.0, 3610.7	
	0	---	---	
Platelet count (1000/uL)	120	1.3	(-1.7,4.4)	0.190
	70	-1.5	(-4.5,1.6)	
	0	---	---	
vWF (ng/mL)	120	-1527.6	-6719.4, 3664.2	0.765
	70	246.3	-4913.4, 5406.0	
	0	---	---	
Fibrinogen (ug/mL)	120	317.3	-67.8, 702.4	0.048
	70	-157.3	-539.9, 225.4	
	0	---	---	

MP-TFA, microparticle-associated tissue factor activity; MP, microparticles; vWF, von Willebrand factor.

^a^Primary outcomes are bolded.

^b^Change from pre- to post-exposure for each ozone concentration, compared to 0 ppb.

**Fig 4:**
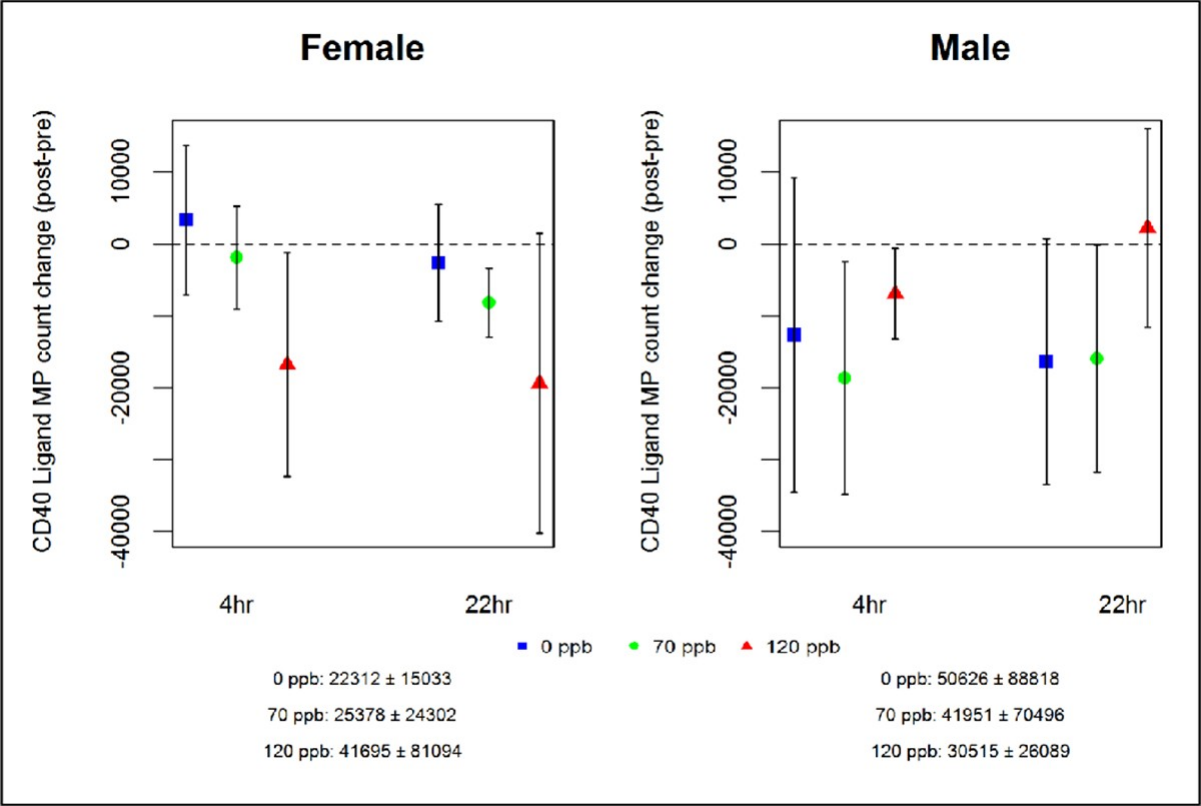
Effect of ozone on CD40 ligand MP count by sex. Changes from pre-exposure across 0, 70, and 120 ppb exposure sessions at 4 h and 22 h post-exposure in females and males.

Ozone exposure did not affect circulating microparticles (MP). There were no significant ozone effects on MP. MP expressing CD40L showed a significant ozone-sex interaction (p<0.001, Table Aaaa in S1 Appendix), with decreases in females and increases in males following ozone, relative to air exposure (Fig 4). There were no significant age or GSTM1 interactions for these outcomes (Tables Yyy, Zzz, Cccc, and Dddd in S1 Appendix).

Ozone exposure did not alter plasma concentrations of vWF or fibrinogen. There were no main ozone effects on vWF (secondary outcome). We observed a marginally significant ozone-age interaction (p = 0.018, Tables Kkkk and Llll in S1 Appendix). For every 1-year increase in age, exposure to 120 ppb ozone resulted in a decrease of 1382 ng/mL in vWF (95% CI, -252, -2512, p = 0.017).

Fibrinogen decreased with 70 ppb ozone (-157 ug/mL) and increased with 120 ppb ozone (317 ug/mL) relative to 0 ppb ozone exposure, but this did not reach statistical significance (p = 0.048). There were no significant age, sex or GSTM1 interactions (Tables Qqqq-Vvvv in S1 Appendix). Fibrinogen levels were 575 μg/mL higher (CI: 6, 1144) in GSTM1 null subjects, independent of ozone exposure.

## Discussion

In this study, we tested the hypothesis that short-term exposure of healthy older adults to near-ambient levels of ozone would induce systemic inflammation leading to endothelial dysfunction and development of a pro-thrombotic vascular state. We chose to study older subjects, postulating that ozone effects would be more likely to lead to acute cardiovascular effects in older than younger subjects. We also postulated that the GSTM1 null genotype would modify responses to ozone exposure. In this panel of 55–70 year-old healthy subjects, we found little evidence of effects of controlled exposure to low levels of ozone on a battery of peripheral venous blood biomarkers of systemic inflammation, oxidative stress, endothelial function, and pro-thrombotic state.

### Systemic inflammation, oxidative stress, and endothelial function

Chronic inflammation has been implicated in the pathogenesis of atherosclerosis and CRP is the inflammatory biomarker that has been most consistently associated with cardiovascular disease risk [42]. It is an acute-phase protein that is produced in the liver in response to pro-inflammatory cytokines, in particular IL-6. It is considered a non-specific marker of inflammation and the mechanism by which CRP contributes to cardiovascular disease risk is unknown.

Several controlled human exposure studies have documented that inhaled ozone at high ambient concentrations can cause airway inflammation characterized by increased neutrophils and pro-inflammatory cytokines in bronchoalveolar lavage or sputum [4, 43]. It is reasonable to suspect that ozone-induced lung inflammation might “spill over” into the systemic circulation, and some data exist to support this concept. A study of pre-exposure levels of serum IL-6 among 45 participants in controlled human exposure studies, in relation to ambient pollutant concentrations in Toronto, reported a positive association between IL-6 and ozone, with the strongest association using a 4-day moving average (~30 ppb) in the summer [44]. Serum CRP increased in two recent controlled human exposure studies in which the ozone concentration ranged from 100–300 ppb [27, 28]. CRP increased after exposure to 200 ppb but not 100 ppb in the Arjomandi et al. study, and only relatively late (18–24 hours) after exposure in both studies. The mean of the pre-exposure levels of CRP in the MOSES study was higher than that reported in those two studies (2.6 mg/L versus 0.6–0.7 mg/L), likely reflecting the older age of our subjects. A sensitivity analysis, using only subjects whose pre-exposure values were below the median of 1.5 mg/L, did not change the results.

Oxidative stress represents an imbalance between the formation of reactive oxygen species in a tissue and that tissue’s antioxidant capacity to detoxify the reactive intermediates. Oxidative stress has been implicated in the pathogenesis of atherosclerosis and has been suggested as a risk factor for adverse cardiovascular outcomes [45]. Ozone is a highly reactive oxidant gas that can increase oxidative stress in the lung. There are both epidemiological and controlled human exposure data to support increased systemic oxidative stress after ozone exposure [46, 47]. 8-isoprostane, a product of lipid peroxidation, has been used as a marker of oxidative stress in previous studies [48, 49].

Based on these data, we hypothesized that ozone exposure would increase serum levels of IL-6, CRP and 8-isoprostane. However, our results showed no evidence of an effect of ozone at either 70 or 120 ppb on these biomarkers of inflammation and oxidative stress. A potential reason for the difference between MOSES results and those of previous controlled human exposure studies is that MOSES subjects were 55–70 years of age while previous studies recruited young adults. Another potential reason is the higher inhaled dose of ozone to which the young adult subjects were exposed in previous studies. It is also possible that our earliest plasma 8-isoprostane measurement, 4 h post-exposure, missed an earlier, transient effect of ozone exposure. However, this time point was selected based on the significant increase reported [46] after exposure of young adults to 200 ppb over 4 h with intermittent exercise. Nonetheless, given that we measured only one biomarker of lipid peroxidation (8-isoprostane), in plasma only (measurement in urine or exhaled breath condensate are alternative approaches), we cannot exclude the possibility of ozone-induced systemic oxidative stress in exercising older subjects.

Nitrotyrosine is a product of tyrosine nitration mediated by reactive nitrogen species such as peroxynitrite anion and NO. Oxidative stress increases the production of superoxide anion and NO, forming peroxynitrite, a highly reactive free radical oxidant capable of oxidizing lipoproteins and nitrating protein tyrosine residues. Nitrotyrosine is easier to measure than peroxynitrite and is used as a marker of NO-dependent, reactive nitrogen species-induced nitrative stress. To our knowledge, MOSES is the first controlled human exposure study of ozone to measure plasma nitrotyrosine. In a mouse model, increased nitrotyrosine residues in the lung after ozone exposure were shown to be an inducible NO synthase (NOSII)-dependent response [50]. We hypothesized that ozone would increase nitrotyrosine; however its concentration surprisingly decreased with increasing ozone concentrations, with marginal statistical significance. As shown in Fig 3B, nitrotyrosine decreased slightly 4 hr after 120 ppb ozone, and decreased relative to 0 ppb with increasing ozone concentrations 22 hr after exposure. However, the only change in which the 95% CI did not include 0 was the increase 22 hr after exposure to 0 ppb ozone. It is possible this increase was spurious, and the relative decreases after 70 and 120 ppb represent regression to the mean. Taken together with the marginal significance and direction of change opposite of that hypothesized, we cannot conclude that there were significant effects of ozone exposure on nitrotyrosine. However, it is possible that reduction in nitrotyrosine levels could be caused by ozone-induced upregulation of antioxidant systems and pathways, quenching reactive nitrative stress.

Endothelin-1 (ET-1) is a potent vasoconstrictor peptide originally isolated from endothelial cells that is now known to be produced by multiple cell types when stimulated, and has been linked to air pollution exposure [51–53]. To our knowledge, ET-1 has not been previously evaluated as an outcome in a controlled human exposure study of ozone. Based on previous reports of the biological effects induced by ET-1 including vasoconstriction, pro-inflammatory responses, mitogenesis, cell proliferation, stimulation of free radical formation, and platelet activation [54], we hypothesized that ozone exposure would increase ET-1. This was confirmed, with a significant overall ozone effect on ET-1 (p = 0.008). While we found no change in ET-1 after 70 ppb at either 4 or 22 h after exposure, we did observe a marginally statistically significant increase after 120 ppb (p = 0.028). Endothelin-1 has important interactions with NO and is involved in the development of endothelial dysfunction. Increased production of ET-1 and its receptors may contribute to the pathogenesis of atherosclerosis [55]. However, as reported elsewhere [35], there was no effect of ozone exposure on blood pressure or forearm flow-mediated dilatation in this study, so the significance of the increase in ET-1 is uncertain. It is possible that the vasoconstrictive effect of increased ET-1 was countered by increases in vasodilators that were not measured, such as NO or prostanoids.

### Prothrombotic vascular status

Increased propensity for vascular thrombosis has been posited as one of the pathways by which exposure to air pollution leads to adverse cardiovascular events. We found no convincing evidence for ozone effects on platelet activation, circulating MP, or MP-TFA. We did find a significant ozone-sex interaction for CD40L+ MP, which decreased in females and increased in males following ozone, compared with air exposure, suggesting this response to ozone exposure may differ by sex. However, none of the other thrombosis markers supported an ozone effect or sex interaction, so we conclude there is no effect on prothrombotic vascular status at these ozone exposure levels in this population.

A few other controlled human exposure studies have examined ozone effects on markers of coagulation or thrombosis, with variable results. Frampton et al. [29] exposed younger, healthy subjects to 0, 100, and 200 ppb ozone for 3 h, with intermittent exercise. There were no significant effects on platelets or circulating microparticles 4 h after exposure, using methods similar to those in the present study. Devlin et al. [28] exposed healthy subjects to 0 and 300 ppb for 2 h with intermittent exercise. They found no effects on plasma levels of vWF or D-dimer. Plasminogen levels increased 24 h after air exposure but decreased after ozone exposure. They also reported a statistically significant increase in tissue plasminogen activator (tPA) and a significant reduction in plasminogen activator inhibitor-1 (PAI-1), the primary inhibitor of tPA, 24 h after ozone exposure, relative to air. These findings indicate possible activation of fibrinolysis following ozone exposure, rather than the hypothesized prothrombotic effect.

Arjomandi et al. [27] found no effects of exposure to 100 and 200 ppb ozone on fibrinogen, PAI-1, prothrombin time, partial thromboplastin time, or platelet count. Similarly, Barath et al. [56] found no effects of 300 ppb ozone for 75 min, with intermittent exercise, on tPA or PAI-1 following brachial arterial infusion of bradykinin, 2 and 6 h after exposure to ozone.

A recent controlled human exposure study examined the effect of varying temperature on the effects of ozone exposure [57]. Healthy volunteers were exposed to 0 and 300 ppb ozone for 2 h with intermittent exercise, at two different ambient temperatures. At 22°C, PAI-1 decreased 52%, plasminogen decreased 12%, and D-dimer increased 18%. In contrast, at 32.5°C, PAI-1 increased 45% and plasminogen increased 28%. D-dimer decreased 12.5%. This suggested that the fibrinolytic pathway is impaired following ozone exposure at the lower temperature and activated at the higher temperature.

A few panel studies have examined the effects of ozone exposure on propensity for thrombosis, but the findings are also mixed. Strak et al. [58] obtained blood from young healthy volunteers at five different locations with varying pollutant concentrations. Intrinsic thrombin generation was associated with increased exposures to nitrogen dioxide, nitrates, and sulfates, but not to ozone. In 76 young, healthy subjects in Taiwan, increased ozone exposure was associated with increased fibrinogen and PAI-1, which held up in two-pollutant models [47]. Liao et al. [59] examined data from the ARIC study that included 10,208 middle-aged people, and found ozone exposure was associated with increases in fibrinogen and vWF levels, with greater effects in people with diabetes and cardiovascular disease.

A weight-of-evidence review [60] described evidence from controlled human exposure studies for small increases in markers of inflammation and oxidative stress in response to ozone exposure, but found no consistent effects on blood coagulation in either clinical or epidemiological studies.

### Strengths and limitations

This multi-center study has a number of strengths. It is one of the largest controlled human exposure studies of ozone to be conducted to date, providing greater statistical power than previous studies. The study was designed to comprehensively evaluate the mechanistic pathways by which air pollutants may contribute to acute cardiovascular toxicity.

The lack of exposure to a higher inhaled dose of ozone limits comparability to the previous controlled exposure studies described above. We cannot therefore generalize the absence of blood biomarker effects to populations exposed to higher concentrations. Also, since we studied acute responses to short-term ozone exposures, we cannot exclude the possibility of effects of chronic or repeated exposures, or effects delayed longer than 22 h after exposure.

The chamber ozone exposure levels of 120 ppb and 70 ppb used in MOSES were only slightly higher than peak ambient ozone levels occurring at the study centers on some days during the study periods. Thus, it is possible that ambient ozone and other pollutant exposures experienced by the study subjects before and during the study may have independently impacted the study biomarkers, and/or modified, blunted, or lessened any biomarker responses to the controlled ozone exposures. These questions are being examined and will be published separately.

By design, we restricted the participants to older, healthy subjects, who were physically fit enough to complete the exercise regimen, and the findings may not be representative of people in other age groups. People with pre-existing cardiovascular disease may differ in their responses to ozone exposure. The choice to study older subjects was based on the hypothesis that these individuals would be at increased risk for acute cardiovascular effects of ozone, but younger individuals are known to be more responsive to the effects of ozone on lung function, and could also be more responsive to cardiovascular effects.

Subject to the same limitations, other data from the MOSES study also failed to reveal significant acute effects on cardiovascular function [35], although there were significant acute changes in respiratory system parameters [34].

## Summary and conclusions

In this multicenter clinical study of older healthy subjects, ozone exposure caused no effects on systemic inflammation. Blood levels of nitrotyrosine decreased with ozone exposure with marginal statistical significance, the opposite of our hypothesis. Blood levels of the potent vasoconstrictor, ET-1, increased with ozone exposure. The results of our study do not support acute effects of low-level ozone exposure in healthy older subjects on systemic inflammation, oxidative stress, or a pro-thrombotic state. We cannot exclude the possibility of effects with higher ozone exposure concentrations or more prolonged exposure, or the possibility that subjects with underlying vascular disease, such as hypertension or diabetes, would show effects under these conditions.

## Supporting information

S1 Appendix Model results for interactions of exposure by age, sex, and GSTM1 status. (DOCX)

S1 Checklist CONSORT 2010 checklist of information to include when reporting a randomised trial. (DOC)

S2 Checklist CONSORT checklist notes. (DOCX)

S1 Protocol Common Protocol for Multicenter Ozone Study in OldEr Subjects (MOSES). (PDF)
