# Supplementary material for: Ozone effects on blood biomarkers of systemic inflammation, oxidative stress, endothelial function, and thrombosis: The Multicenter Ozone Study in oldEr Subjects (MOSES)
Source: PLoS One. 2019 Sep 25;14(9):e0222601. doi: 10.1371/journal.pone.0222601 (PMC6760801; doi:10.1371/journal.pone.0222601)
Supplement: S1 Appendix — (DOCX) [file pone.0222601.s001.docx]

**S1 Appendix**

**Model results for interactions of exposure by age, sex, and glutathione S-transferase Mu 1 gene (GSTM1) status**

**Table A. Type III sum of squares for change in CRP (mg/L) including ozone exposure by age interaction.**

| Effect | P-value |
| --- | --- |
| Ozone exposure | 0.6551 |
| 4 h vs. 22 h change | 0.1810 |
| Site | 0.1660 |
| Age | 0.5199 |
| Ozone exposure by age | 0.8129 |

CRP, c-reactive protein.

**Table B. Mixed model for change in CRP (mg/L) including ozone exposure by age interaction.**

| Effect | Estimate | Lower  95% CI | Upper  95% CI | P-value |
| --- | --- | --- | --- | --- |
| Intercept | -0.16 | -0.68 | 0.36 | 0.5505 |
| 4 h change | -0.21 | -0.53 | 0.10 | 0.1810 |
| 22 h change | 0 | --- | --- | --- |
| Ozone exposure |  |  |  |  |
| 120 ppb | -0.16 | -0.54 | 0.23 | 0.4196 |
| 70 ppb | -0.15 | -0.54 | 0.23 | 0.4330 |
| 0 ppb | 0 | --- | --- | --- |
| Site |  |  |  |  |
| URMC | 0.44 | -0.16 | 1.04 | 0.1479 |
| UNC | -0.09 | -0.70 | 0.53 | 0.7764 |
| UCSF | 0 | --- | --- | --- |
| Age | -0.03 | -0.10 | 0.05 | 0.4636 |
| Ozone exposure by age |  |  |  |  |
| 120 ppb by age | 0.03 | -0.06 | 0.11 | 0.5546 |
| 70 ppb by age | 0.00 | -0.08 | 0.09 | 0.9367 |
| 0 ppb by age | 0 | --- | --- | --- |

CRP, c-reactive protein; URMC, University of Rochester Medical Center; UNC, University of North Carolina; UCSF, University of California San Francisco.

**Table C. Type III sum of squares for change in CRP (mg/L) including ozone exposure by sex interaction.**

| Effect | P-value |
| --- | --- |
| Ozone exposure | 0.5228 |
| 4 h vs. 22 h change | 0.1782 |
| Site | 0.1998 |
| Sex | 0.4172 |
| Ozone exposure by sex | 0.1649 |

CRP, c-reactive protein.

**Table D. Mixed model for change in CRP (mg/L) including ozone exposure by sex interaction.**

| Effect | Estimate | Lower  95% CI | Upper  95% CI | P-value |
| --- | --- | --- | --- | --- |
| Intercept | -0.06 | -0.69 | 0.56 | 0.8430 |
| 4 h change | -0.21 | -0.53 | 0.10 | 0.1782 |
| 22 h change | 0 | --- | --- | --- |
| Ozone exposure |  |  |  |  |
| 120 ppb | -0.58 | -1.17 | 0.01 | 0.0548 |
| 70 ppb | -0.29 | -0.89 | 0.30 | 0.3319 |
| 0 ppb | 0 | --- | --- | --- |
| Site |  |  |  |  |
| URMC | 0.38 | -0.22 | 0.98 | 0.2070 |
| UNC | -0.12 | -0.75 | 0.50 | 0.6935 |
| UCSF | 0 | --- | --- | --- |
| Sex |  |  |  |  |
| Female | -0.12 | -0.80 | 0.56 | 0.7262 |
| Male | 0 | --- | --- | --- |
| Ozone exposure by sex |  |  |  |  |
| 120 ppb by female | 0.74 | -0.04 | 1.52 | 0.0631 |
| 70 ppb by female | 0.24 | -0.54 | 1.02 | 0.5439 |
| 0 ppb by female | 0 | --- | --- | --- |
| 120 ppb by male | 0 | --- | --- | --- |
| 70 ppb by male | 0 | --- | --- | --- |
| 0 ppb by male | 0 | --- | --- | --- |

CRP, c-reactive protein; URMC, University of Rochester Medical Center; UNC, University of North Carolina; UCSF, University of California San Francisco.

**Table E. Type III sum of squares for change in CRP (mg/L) including ozone exposure by GSTM1 status interaction.**

| Effect | P-value |
| --- | --- |
| Ozone exposure | 0.7214 |
| 4 h vs. 22 h change | 0.1834 |
| Site | 0.1830 |
| GSTM1 status | 0.7974 |
| Ozone exposure by GSTM1 status | 0.6761 |

CRP, c-reactive protein; GSTM1, glutathione S-transferase Mu 1 gene.

**Table F. Mixed model for change in CRP (mg/L) including ozone exposure by GSTM1 status interaction.**

| Effect | Estimate | Lower  95% CI | Upper  95% CI | P-value |
| --- | --- | --- | --- | --- |
| Intercept | -0.08 | -0.66 | 0.50 | 0.7901 |
| 4 h change | -0.21 | -0.53 | 0.10 | 0.1834 |
| 22 h change | 0 | --- | --- | --- |
| Ozone exposure |  |  |  |  |
| 120 ppb | -0.29 | -0.80 | 0.21 | 0.2535 |
| 70 ppb | -0.19 | -0.69 | 0.32 | 0.4683 |
| 0 ppb | 0 | --- | --- | --- |
| Site |  |  |  |  |
| URMC | 0.43 | -0.17 | 1.03 | 0.1593 |
| UNC | -0.08 | -0.70 | 0.54 | 0.7960 |
| UCSF | 0 |  |  |  |
| GSTM1 status |  |  |  |  |
| Sufficient | -0.20 | -0.87 | 0.47 | 0.5566 |
| Null | 0 | --- | --- | --- |
| Ozone exposure by GSTM1 status |  |  |  |  |
| 120 ppb by sufficient | 0.33 | -0.45 | 1.12 | 0.4012 |
| 70 ppb by sufficient | 0.07 | -0.70 | 0.85 | 0.8534 |
| 0 ppb by sufficient | 0 | --- | --- | --- |
| 120 ppb by null | 0 | --- | --- | --- |
| 70 ppb by null | 0 | --- | --- | --- |
| 0 ppb by null | 0 | --- | --- | --- |

CRP, c-reactive protein; GSTM1, glutathione S-transferase Mu 1 gene; URMC, University of Rochester Medical Center; UNC, University of North Carolina; UCSF, University of California San Francisco.

**Table G. Type III sum of squares for change in interleukin-6 (pg/mL) including ozone exposure by age interaction.**

| Effect | P-value |
| --- | --- |
| Ozone exposure | 0.5692 |
| 4 h vs. 22 h change | 0.3899 |
| Site | 0.8027 |
| Age | 0.6143 |
| Ozone exposure by age | 0.9843 |

**Table H. Mixed model for change in IL-6 (pg/mL) including ozone exposure by age interaction.**

| Effect | Estimate | Lower  95% CI | Upper  95% CI | P-value |
| --- | --- | --- | --- | --- |
| Intercept | 0.00 | -0.62 | 0.63 | 0.9899 |
| 4 h change | -0.18 | -0.60 | 0.24 | 0.3899 |
| 22 h change | 0 | --- | --- | --- |
| Ozone exposure |  |  |  |  |
| 120 ppb | -0.22 | -0.74 | 0.29 | 0.3862 |
| 70 ppb | -0.25 | -0.76 | 0.26 | 0.3354 |
| 0 ppb | 0 | --- | --- | --- |
| Site |  |  |  |  |
| URMC | 0.18 | -0.50 | 0.87 | 0.5955 |
| UNC | 0.22 | -0.49 | 0.92 | 0.5392 |
| UCSF | 0 | --- | --- | --- |
| Age | 0.02 | -0.07 | 0.11 | 0.6606 |
| Ozone exposure by age |  |  |  |  |
| 120 ppb by age | -0.00 | -0.11 | 0.11 | 0.9597 |
| 70 ppb by age | -0.01 | -0.12 | 0.10 | 0.8630 |
| 0 ppb by age | 0 | --- | --- | --- |

IL-6, interleukin-6; URMC, University of Rochester Medical Center; UNC, University of North Carolina; UCSF, University of California San Francisco.

**Table I. Type III sum of squares for change in interleukin-6 (pg/mL) including ozone exposure by sex interaction.**

| Effect | P-value |
| --- | --- |
| Ozone exposure | 0.3285 |
| 4 h vs. 22 h change | 0.3937 |
| Site | 0.8791 |
| Sex | 0.4178 |
| Ozone exposure by sex | 0.0276 |

**Table J. Mixed model for change in IL-6 (pg/mL) including ozone exposure by sex interaction.**

| Effect | Estimate | Lower  95% CI | Upper  95% CI | P-value |
| --- | --- | --- | --- | --- |
| Intercept | 0.26 | -0.49 | 1.02 | 0.4884 |
| 4 h change | -0.18 | -0.59 | 0.24 | 0.3937 |
| 22 h change | 0 | --- | --- | --- |
| Ozone exposure |  |  |  |  |
| 120 ppb | -0.52 | -1.30 | 0.26 | 0.1879 |
| 70 ppb | -1.07 | -1.85 | -0.29 | 0.0078 |
| 0 ppb | 0 | --- | --- | --- |
| Site |  |  |  |  |
| URMC | 0.15 | -0.53 | 0.84 | 0.6593 |
| UNC | 0.16 | -0.55 | 0.87 | 0.6545 |
| UCSF | 0 | --- | --- | --- |
| Sex |  |  |  |  |
| Female | -0.40 | -1.22 | 0.43 | 0.3428 |
| Male | 0 | --- | --- | --- |
| Ozone exposure by sex |  |  |  |  |
| 120 ppb by female | 0.51 | -0.51 | 1.54 | 0.3233 |
| 70 ppb by female | 1.38 | 0.36 | 2.41 | 0.0081 |
| 0 ppb by female | 0 | --- | --- | --- |
| 120 ppb by male | 0 | --- | --- | --- |
| 70 ppb by male | 0 | --- | --- | --- |
| 0 ppb by male | 0 | --- | --- | --- |

IL-6, interleukin-6; URMC, University of Rochester Medical Center; UNC, University of North Carolina; UCSF, University of California San Francisco.

**Table K. Type III sum of squares for change in IL-6 (pg/mL) including ozone exposure by GSTM1 status interaction.**

| Effect | P-value |
| --- | --- |
| Ozone exposure | 0.7639 |
| 4 h vs. 22 h change | 0.4040 |
| Site | 0.7184 |
| GSTM1 status | 0.1544 |
| Ozone exposure by GSTM1 status | 0.0310 |

IL-6, interleukin-6; GSTM1, glutathione S-transferase Mu 1 gene.

**Table L. Mixed model for change in IL-6 (pg/mL) including ozone exposure by GSTM1 status interaction.**

| Effect | Estimate | Lower  95% CI | Upper  95% CI | P-value |
| --- | --- | --- | --- | --- |
| Intercept | 0.39 | -0.30 | 1.08 | 0.2647 |
| 4 h change | -0.17 | -0.59 | 0.24 | 0.4040 |
| 22 h change | 0 | --- | --- | --- |
| Ozone exposure |  |  |  |  |
| 120 ppb | -0.41 | -1.07 | 0.25 | 0.2202 |
| 70 ppb | -0.81 | -1.47 | -0.15 | 0.0160 |
| 0 ppb | 0 | --- | --- | --- |
| Site |  |  |  |  |
| URMC | 0.25 | -0.43 | 0.93 | 0.4668 |
| UNC | 0.24 | -0.46 | 0.94 | 0.4896 |
| UCSF | 0 | --- | --- | --- |
| GSTM1 status |  |  |  |  |
| Sufficient | -1.01 | -1.83 | -0.19 | 0.0166 |
| Null | 0 |  |  |  |
| Ozone exposure by GSTM1 status |  |  |  |  |
| 120 ppb by sufficient | 0.46 | -0.57 | 1.48 | 0.3814 |
| 70 ppb by sufficient | 1.35 | 0.33 | 2.37 | 0.0098 |
| 0 ppb by sufficient | 0 | --- | --- | --- |
| 120 ppb by null | 0 | --- | --- | --- |
| 70 ppb by null | 0 | --- | --- | --- |
| 0 ppb by null | 0 | --- | --- | --- |

IL-6, interleukin-6; GSTM1, glutathione S-transferase Mu 1 gene; URMC, University of Rochester Medical Center; UNC, University of North Carolina; UCSF, University of California San Francisco.

**Table M. Type III sum of squares for change in 8-isoprostane (pg/mL) including ozone exposure by age interaction.**

| Effect | P-value |
| --- | --- |
| Ozone exposure | 0.6837 |
| 4hr v 22hr change | 0.1371 |
| Site | 0.6655 |
| Age | 0.6480 |
| Ozone exposure by age | 0.1140 |

**Table N. Mixed model for change in 8-isoprostane (pg/mL) including ozone exposure by age interaction.**

| Effect | Estimate | Lower  95% CI | Upper  95% CI | P-value |
| --- | --- | --- | --- | --- |
| Intercept | -1.39 | -7.37 | 4.59 | 0.6454 |
| 4hr change | -3.07 | -7.13 | 1.00 | 0.1371 |
| 22hr change | 0 | --- | --- | --- |
| Ozone exposure |  |  |  |  |
| 120 ppb | -0.81 | -5.78 | 4.17 | 0.7494 |
| 70 ppb | -2.16 | -7.10 | 2.78 | 0.3892 |
| 0 ppb | 0 | --- | --- | --- |
| Site |  |  |  |  |
| URMC | 2.95 | -3.61 | 9.52 | 0.3731 |
| UNC | 1.22 | -5.52 | 7.95 | 0.7198 |
| UCSF | 0 | --- | --- | --- |
| Age | -0.35 | -1.22 | 0.52 | 0.4286 |
| Ozone exposure by age |  |  |  |  |
| 120 ppb by age | -0.23 | -1.31 | 0.86 | 0.6836 |
| 70 ppb by age | 0.86 | -0.22 | 1.95 | 0.1189 |
| 0 ppb by age | 0 | --- | --- | --- |

URMC, University of Rochester Medical Center; UNC, University of North Carolina; UCSF, University of California San Francisco.

**Table O. Type III sum of squares for change in 8-isoprostane (pg/mL) including ozone exposure by sex interaction.**

| Effect | P-value |
| --- | --- |
| Ozone exposure | 0.5967 |
| 4 hr v 22 hr change | 0.1367 |
| Site | 0.6848 |
| Sex | 0.8741 |
| Ozone exposure by sex | 0.1335 |

**Table P. Mixed model for change in 8-isoprostane (pg/mL) including ozone exposure by sex interaction.**

| Effect | Estimate | Lower  95% CI | Upper  95% CI | P-value |
| --- | --- | --- | --- | --- |
| Intercept | -1.01 | -8.33 | 6.31 | 0.7852 |
| 4 hr change | -3.07 | -7.13 | 0.99 | 0.1367 |
| 22 hr change | 0 | --- | --- | --- |
| Ozone exposure |  |  |  |  |
| 120 ppb | 1.80 | -5.84 | 9.45 | 0.6421 |
| 70 ppb | -5.26 | -12.97 | 2.45 | 0.1801 |
| 0 ppb | 0 | --- | --- | --- |
| Site |  |  |  |  |
| URMC | 2.87 | -3.72 | 9.46 | 0.3887 |
| UNC | 1.32 | -5.51 | 8.16 | 0.7016 |
| UCSF | 0 | --- | --- | --- |
| Sex |  |  |  |  |
| Female | -0.75 | -8.79 | 7.28 | 0.8525 |
| Male | 0 | --- | --- | --- |
| Ozone exposure by sex |  |  |  |  |
| 120 ppb by female | -4.66 | -14.72 | 5.40 | 0.3617 |
| 70 ppb by female | 5.60 | -4.44 | 15.64 | 0.2724 |
| 0 ppb by female | 0 | --- | --- | --- |
| 120 ppb by male | 0 | --- | --- | --- |
| 70 ppb by male | 0 | --- | --- | --- |
| 0 ppb by male | 0 | --- | --- | --- |

URMC, University of Rochester Medical Center; UNC, University of North Carolina; UCSF, University of California San Francisco.

**Table Q. Type III sum of squares for change in 8-isoprostane (pg/mL) including ozone exposure by GSTM1 status interaction.**

| Effect | P-value |
| --- | --- |
| Ozone exposure | 0.7962 |
| 4hr v 22hr change | 0.1388 |
| Site | 0.6384 |
| GSTM1 status | 0.4684 |
| Ozone exposure by GSTM1 status | 0.6427 |

GSTM1, glutathione S-transferase Mu 1 gene

**Table R. Mixed model for change in 8-isoprostane (pg/mL) including ozone exposure by GSTM1 status interaction.**

| Effect | Estimate | Lower  95% CI | Upper  95% CI | P-value |
| --- | --- | --- | --- | --- |
| Intercept | 0.28 | -6.43 | 6.99 | 0.9346 |
| 4hr change | -3.07 | -7.14 | 1.01 | 0.1388 |
| 22hr change | 0 | --- | --- | --- |
| Ozone exposure |  |  |  |  |
| 120 ppb | -2.88 | -9.39 | 3.64 | 0.3845 |
| 70 ppb | -2.98 | -9.48 | 3.52 | 0.3662 |
| 0 ppb | 0 | --- | --- | --- |
| Site |  |  |  |  |
| URMC | 3.11 | -3.43 | 9.65 | 0.3470 |
| UNC | 1.45 | -5.28 | 8.18 | 0.6699 |
| UCSF | 0 | --- | --- | --- |
| GSTM1 status |  |  |  |  |
| Sufficient | -4.48 | -12.49 | 3.54 | 0.2697 |
| Null | 0 | --- | --- | --- |
| Ozone exposure by GSTM1 status |  |  |  |  |
| 120 ppb by sufficient | 4.82 | -5.31 | 14.95 | 0.3485 |
| 70 ppb by sufficient | 2.64 | -7.41 | 12.68 | 0.6052 |
| 0 ppb by sufficient | 0 | --- | --- | --- |
| 120 ppb by null | 0 | --- | --- | --- |
| 70 ppb by null | 0 | --- | --- | --- |
| 0 ppb by null | 0 | --- | --- | --- |

GSTM1, glutathione S-transferase Mu 1 gene; URMC, University of Rochester Medical Center; UNC, University of North Carolina; UCSF, University of California San Francisco.

**Table S. Type III sum of squares for change in P-selectin (ng/mL) including ozone exposure by age interaction.**

| Effect | P-value |
| --- | --- |
| Ozone exposure | 0.2665 |
| 4 hr vs. 22 hr change | 0.0074 |
| Site | 0.2099 |
| Age | 0.3468 |
| Ozone exposure by age | 0.0956 |

**Table T. Mixed model for change in P-selectin (ng/mL) including ozone exposure by age interaction.**

| Effect | Estimate | Lower 95% CI | Upper 95% CI | P-value |
| --- | --- | --- | --- | --- |
| Intercept | 55.66 | 22.39 | 88.92 | 0.0013 |
| 4 hr change | -31.85 | -54.93 | -8.77 | 0.0074 |
| 22 hr change | 0 | --- | --- | --- |
| Ozone exposure |  |  |  |  |
| 120 ppb | -13.17 | -41.42 | 15.08 | 0.3586 |
| 70 ppb | -23.17 | -51.26 | 4.92 | 0.1054 |
| 0 ppb | 0 | --- | --- | --- |
| Site |  |  |  |  |
| URMC | -28.21 | -64.29 | 7.86 | 0.1236 |
| UNC | -29.03 | -66.06 | 8.01 | 0.1228 |
| UCSF | 0 | --- | --- | --- |
| Age | 5.50 | 0.61 | 10.40 | 0.0280 |
| Ozone exposure by age |  |  |  |  |
| 120 ppb by age | -5.78 | -11.96 | 0.41 | 0.0670 |
| 70 ppb by age | -6.06 | -12.23 | 0.11 | 0.0543 |
| 0 ppb by age | 0 | --- | --- | --- |

URMC, University of Rochester Medical Center; UNC, University of North Carolina; UCSF, University of California San Francisco.

**Table U. Type III sum of squares for change in P-selectin (ng/mL) including ozone exposure by sex interaction.**

| Effect | P-value |
| --- | --- |
| Ozone exposure | 0.1783 |
| 4 hr vs. 22 hr change | 0.0078 |
| Site | 0.2360 |
| Sex | 0.9438 |
| Ozone exposure by sex | 0.3245 |

**Table V. Mixed model for change in P-selectin (ng/mL) including ozone exposure by sex interaction.**

| Effect | Estimate | Lower 95% CI | Upper 95% CI | P-value |
| --- | --- | --- | --- | --- |
| Intercept | 70.43 | 29.45 | 111.41 | 0.0010 |
| 4 h change | -31.76 | -54.92 | -8.61 | 0.0078 |
| 22 h change | 0 | --- | --- | --- |
| Ozone exposure |  |  |  |  |
| 120 ppb | -38.85 | -82.42 | 4.71 | 0.0801 |
| 70 ppb | -40.95 | -84.88 | 2.99 | 0.0676 |
| 0 ppb | 0 | --- | --- | --- |
| Site |  |  |  |  |
| URMC | -26.67 | -63.01 | 9.66 | 0.1481 |
| UNC | -29.28 | -66.99 | 8.43 | 0.1263 |
| UCSF | 0 | --- | --- | --- |
| Sex |  |  |  |  |
| Female | -24.93 | -70.04 | 20.18 | 0.2749 |
| Male | 0 | --- | --- | --- |
| Ozone exposure by sex |  |  |  |  |
| 120 ppb by female | 42.91 | -14.42 | 100.24 | 0.1414 |
| 70 ppb by female | 28.63 | -28.58 | 85.83 | 0.3246 |
| 0 ppb by female | 0 | --- | --- | --- |
| 120 ppb by male | 0 | --- | --- | --- |
| 70 ppb by male | 0 | --- | --- | --- |
| 0 ppb by male | 0 | --- | --- | --- |

URMC, University of Rochester Medical Center; UNC, University of North Carolina; UCSF, University of California San Francisco.

**Table W. Type III sum of squares for change in P-selectin (ng/mL) including ozone exposure by GSTM1 status interaction.**

| Effect | P-value |
| --- | --- |
| Ozone exposure | 0.3319 |
| 4 hr vs. 22 hr change | 0.0081 |
| Site | 0.2571 |
| GSTM1 status | 0.2691 |
| Ozone exposure by GSTM1 status | 0.5017 |

GSTM1, glutathione S-transferase Mu 1 gene.

**Table X. Mixed model for change in P-selectin (ng/mL) including ozone exposure by GSTM1 status interaction.**

| Effect | Estimate | Lower 95% CI | Upper 95% CI | P-value |
| --- | --- | --- | --- | --- |
| Intercept | 68.38 | 31.00 | 105.75 | 0.0005 |
| 4 hr change | -31.62 | -54.80 | -8.43 | 0.0081 |
| 22 hr change | 0 | --- | --- | --- |
| Ozone exposure |  |  |  |  |
| 120 ppb | -19.70 | -56.73 | 17.32 | 0.29 |
| 70 ppb | -38.33 | -75.25 | -1.41 | 0.04 |
| 0 ppb | 0 | --- | --- | --- |
| Site |  |  |  |  |
| URMC | -24.65 | -60.57 | 11.27 | 0.1760 |
| UNC | -28.42 | -65.39 | 8.56 | 0.1302 |
| UCSF | 0 | --- | --- | --- |
| GSTM1 status |  |  |  |  |
| Sufficient | -32.55 | -77.38 | 12.29 | 0.1526 |
| Null | 0 | --- | --- | --- |
| Ozone exposure by GSTM1 status |  |  |  |  |
| 120 ppb by sufficient | 13.76 | -43.80 | 71.32 | 0.64 |
| 70 ppb by sufficient | 33.81 | -23.28 | 90.90 | 0.24 |
| 0 ppb by sufficient | 0 | --- | --- | --- |
| 120 ppb by null | 0 | --- | --- | --- |
| 70 ppb by null | 0 | --- | --- | --- |
| 0 ppb by null | 0 | --- | --- | --- |

GSTM1, glutathione S-transferase Mu 1 gene; URMC, University of Rochester Medical Center; UNC, University of North Carolina; UCSF, University of California San Francisco.

**Table Y. Type III sum of squares for change in nitrotyrosine (nM) including ozone exposure by age interaction.**

| Effect | P-value |
| --- | --- |
| Ozone exposure | 0.0169 |
| 4 h vs. 22 h change | 0.4808 |
| Site | 0.6215 |
| Age | 0.7412 |
| Ozone exposure by age | 0.6570 |

**Table Z. Mixed model for change in nitrotyrosine (nM) including ozone exposure by age interaction.**

| Effect | Estimate | Lower  95% CI | Upper  95% CI | P-value |
| --- | --- | --- | --- | --- |
| Intercept | 41.2 | 3.8 | 79.0 | 0.0313 |
| 4 h change | -8.4 | -31.9 | 15.1 | 0.4808 |
| 22 h change | 0 | --- | --- | --- |
| Ozone exposure |  |  |  |  |
| 120 ppb | -41.6 | -70.3 | -12.9 | 0.0048 |
| 70 ppb | -14.9 | -43.5 | 13.6 | 0.3032 |
| 0 ppb | 0 | --- | --- | --- |
| Site |  |  |  |  |
| URMC | -8.1 | -50.7 | 34.5 | 0.7057 |
| UNC | -21.2 | -65.0 | 22.5 | 0.3368 |
| UCSF | 0 | --- | --- | --- |
| Age | -2.0 | -7.4 | 3.3 | 0.4535 |
| Ozone exposure by age |  |  |  |  |
| 120 ppb by age | 1.2 | -5.1 | 7.5 | 0.7005 |
| 70 ppb by age | 2.9 | -3.3 | 9.2 | 0.3619 |
| 0 ppb by age | 0 | --- | --- | --- |

URMC, University of Rochester Medical Center; UNC, University of North Carolina; UCSF, University of California San Francisco.

**Table Aa. Type III sum of squares for change in nitrotyrosine (nM) including ozone exposure by sex interaction.**

| Effect | P-value |
| --- | --- |
| Ozone exposure | 0.0085 |
| 4 h vs. 22 h change | 0.4819 |
| Site | 0.6895 |
| Sex | 0.6693 |
| Ozone exposure by sex | 0.1828 |

**Table Bb. Mixed model for change in nitrotyrosine (nM) including ozone exposure by sex interaction.**

| Effect | Estimate | Lower  95% CI | Upper  95% CI | P-value |
| --- | --- | --- | --- | --- |
| Intercept | 61.9 | 16.6 | 107.1 | 0.0079 |
| 4 h change | -8.3 | -31.7 | 15.1 | 0.4819 |
| 22 h change | 0 | --- | --- | --- |
| Ozone exposure |  |  |  |  |
| 120 ppb | -72.1 | -116.0 | -28.1 | 0.0015 |
| 70 ppb | -35.5 | -79.9 | 8.8 | 0.1157 |
| 0 ppb | 0 | --- | --- | --- |
| Site |  |  |  |  |
| URMC | -7.7 | -50.4 | 35.1 | 0.7222 |
| UNC | -19.1 | -63.4 | 25.3 | 0.3951 |
| UCSF | 0 | --- | --- | --- |
| Sex |  |  |  |  |
| Female | -37.7 | -86.8 | 11.5 | 0.1312 |
| Male | 0 | --- | --- | --- |
| Ozone exposure by sex |  |  |  |  |
| 120 ppb by female | 53.1 | -4.9 | 111.0 | 0.0723 |
| 70 ppb by female | 36.7 | -21.1 | 94.5 | 0.2116 |
| 0 ppb by female | 0 | --- | --- | --- |
| 120 ppb by male | 0 | --- | --- | --- |
| 70 ppb by male | 0 | --- | --- | --- |
| 0 ppb by male | 0 | --- | --- | --- |

URMC, University of Rochester Medical Center; UNC, University of North Carolina; UCSF, University of California San Francisco.

**Table Cc. Type III sum of squares for change in nitrotyrosine (nM) including ozone exposure by GSTM1 status interaction.**

| Effect | P-value |
| --- | --- |
| Ozone exposure | 0.0138 |
| 4 h vs. 22 h change | 0.4827 |
| Site | 0.6415 |
| GSTM1 status | 0.7818 |
| Ozone exposure by GSTM1 status | 0.2605 |

GSTM1, glutathione S-transferase Mu 1 gene.

**Table Dd. Mixed model for change in nitrotyrosine (nM) including ozone exposure by GSTM1 status interaction.**

| Effect | Estimate | Lower  95% CI | Upper  95% CI | P-value |
| --- | --- | --- | --- | --- |
| Intercept | 46.4 | 4.8 | 88.1 | 0.0293 |
| 4 h change | -8.3 | -31.7 | 15.1 | 0.4827 |
| 22 h change | 0 | --- | --- | --- |
| Ozone exposure |  |  |  |  |
| 120 ppb | -37.5 | -75.0 | -0.1 | 0.0497 |
| 70 ppb | -29.4 | -66.7 | 7.9 | 0.1215 |
| 0 ppb | 0 | --- | --- | --- |
| Site |  |  |  |  |
| URMC | -8.3 | -50.8 | 34.2 | 0.6995 |
| UNC | -20.6 | -64.3 | 23.2 | 0.3523 |
| UCSF | 0 | --- | --- | --- |
| GSTM1 status | -13.8 | -62.6 | 35.1 | 0.5767 |
| Sufficient | 0 | --- | --- | --- |
| Null |  |  |  |  |
| Ozone exposure by GSTM1 status |  |  |  |  |
| 120 ppb by sufficient | -9.5 | -67.7 | 48.7 | 0.7474 |
| 70 ppb by sufficient | 36.0 | -21.7 | 93.7 | 0.2194 |
| 0 ppb by sufficient | 0 | --- | --- | --- |
| 120 ppb by null | 0 | --- | --- | --- |
| 70 ppb by null | 0 | --- | --- | --- |
| 0 ppb by null | 0 | --- | --- | --- |

GSTM1, glutathione S-transferase Mu 1 gene; URMC, University of Rochester Medical Center; UNC, University of North Carolina; UCSF, University of California San Francisco.

**Table Ee. Type III sum of squares for change in nitrotyrosine (nM) including ozone exposure by time interaction.**

| Effect | P-value |
| --- | --- |
| 4 h vs. 22 h change | 0.6332 |
| Ozone exposure | 0.0046 |
| Site | 0.7616 |
| Ozone exposure by time | 0.5101 |

**Table Ff. Mixed model for change in nitrotyrosine (nM) including ozone exposure by time interaction.**

| Effect | Estimate | Lower  95% CI | Upper  95% CI | P-value |
| --- | --- | --- | --- | --- |
| Intercept | -4.3 | -66.8 | 58.2 | 0.8926 |
| 22 h change | 38.7 | -25.3 | 102.8 | 0.2328 |
| 4 h change | 0 | --- | --- | --- |
| Ozone exposure |  |  |  |  |
| 120 ppb | -52.5 | -115.8 | 10.8 | 0.1034 |
| 70 ppb | 10.4 | -52.7 | 73.5 | 0.7459 |
| 0 ppb | 0 | --- | --- | --- |
| Site |  |  |  |  |
| URMC | 23.6 | -40.1 | 87.2 | 0.4640 |
| UNC | 10.9 | -54.8 | 76.6 | 0.7430 |
| UCSF | 0 | --- | --- | --- |
| Ozone exposure by time |  |  |  |  |
| 120 ppb | -39.3 | -129.4 | 50.9 | 0.3908 |
| 70 ppb | -50.3 | -139.8 | 39.3 | 0.2694 |
| 0 ppb | 0 | --- | --- | --- |

URMC, University of Rochester Medical Center; UNC, University of North Carolina; UCSF, University of California San Francisco.

**Table Gg. Type III sum of squares for change in endothelin-1 (pg/mL) including ozone exposure by age interaction.**

| Effect | P-value |
| --- | --- |
| Ozone exposure | 0.0101 |
| 4 hr vs. 22 hr change | 0.2581 |
| Site | 0.9393 |
| Age | 0.0405 |
| Ozone exposure by age | 0.7610 |

**Table Hh. Mixed model for change in endothelin-1 (pg/mL) including ozone exposure by age interaction.**

| Effect | Estimate | Lower 95% CI | Upper 95% CI | P-value |
| --- | --- | --- | --- | --- |
| Intercept | -0.04 | -0.12 | 0.05 | 0.3629 |
| 4 hr change | 0.03 | -0.02 | 0.09 | 0.2581 |
| 22 hr change | 0 | --- | --- | --- |
| Ozone exposure |  |  |  |  |
| 120 ppb | 0.07 | 0.01 | 0.14 | 0.0292 |
| 70 ppb | -0.03 | -0.09 | 0.04 | 0.4503 |
| 0 ppb | 0 | --- | --- | --- |
| Site |  |  |  |  |
| URMC | -0.02 | -0.11 | 0.08 | 0.7281 |
| UNC | -0.01 | -0.11 | 0.09 | 0.8150 |
| UCSF | 0 | --- | --- | --- |
| Age | -0.01 | -0.02 | 0.00 | 0.1621 |
| Ozone exposure by age |  |  |  |  |
| 120 ppb by age | 0.00 | -0.01 | 0.02 | 0.7721 |
| 70 ppb by age | -0.00 | -0.02 | 0.01 | 0.6599 |
| 0 ppb by age | 0 | --- | --- | --- |

URMC, University of Rochester Medical Center; UNC, University of North Carolina; UCSF, University of California San Francisco.

**Table Ii. Type III sum of squares for change in endothelin-1 (pg/mL) including ozone exposure by sex interaction.**

| Effect | P-value |
| --- | --- |
| Ozone exposure | 0.0050 |
| 4 hr vs. 22 hr change | 0.2615 |
| Site | 0.8006 |
| Sex | 0.4107 |
| Ozone exposure by sex | 0.1022 |

**Table Jj. Mixed model for change in endothelin-1 (pg/mL) including ozone exposure by sex interaction.**

| Effect | Estimate | Lower 95% CI | Upper 95% CI | P-value |
| --- | --- | --- | --- | --- |
| Intercept | -0.10 | -0.20 | 0.01 | 0.0621 |
| 4 hr change | 0.03 | -0.02 | 0.08 | 0.2615 |
| 22 hr change | 0 | --- | --- | --- |
| Ozone exposure |  |  |  |  |
| 120 ppb | 0.16 | 0.055 | 0.26 | 0.0027 |
| 70 ppb | 0.03 | -0.08 | 0.13 | 0.6013 |
| 0 ppb | 0 | --- | --- | --- |
| Site |  |  |  |  |
| URMC | -0.03 | -0.13 | 0.06 | 0.5062 |
| UNC | -0.02 | -0.12 | 0.08 | 0.7260 |
| UCSF | 0 | --- | --- | --- |
| Sex |  |  |  |  |
| Female | 0.11 | 0.00 | 0.22 | 0.0483 |
| Male | 0 | --- | --- | --- |
| Ozone exposure by sex |  |  |  |  |
| 120 ppb by female | -0.14 | -0.28 | -0.01 | 0.0361 |
| 70 ppb by female | -0.09 | -0.23 | 0.04 | 0.1619 |
| 0 ppb by female | 0 | --- | --- | --- |
| 120 ppb by male | 0 | --- | --- | --- |
| 70 ppb by male | 0 | --- | --- | --- |
| 0 ppb by male | 0 | --- | --- | --- |

URMC, University of Rochester Medical Center; UNC, University of North Carolina; UCSF, University of California San Francisco.

**Table Kk. Type III sum of squares for change in endothelin-1 (pg/mL) including ozone exposure by GSTM1 status interaction.**

| Effect | P-value |
| --- | --- |
| Ozone exposure | 0.0068 |
| 4 hr vs. 22 hr change | 0.2606 |
| Site | 0.7995 |
| GSTM1 status | 0.3628 |
| Ozone exposure by GSTM1 status | 0.8030 |

GSTM1, glutathione S-transferase Mu 1 gene.

**Table Ll. Mixed model for change in endothelin-1 (pg/mL) including ozone exposure by GSTM1 status interaction**

| Effect | Estimate | Lower 95% CI | Upper 95% CI | P-value |
| --- | --- | --- | --- | --- |
| Intercept | -0.04 | -0.14 | 0.05 | 0.3697 |
| 4 hr change | 0.03 | -0.02 | 0.09 | 0.2606 |
| 22 hr change | 0 | --- | --- | --- |
| Ozone exposure |  |  |  |  |
| 120 ppb | 0.06 | -0.03 | 0.14 | 0.1954 |
| 70 ppb | -0.03 | -0.12 | 0.06 | 0.4966 |
| 0 ppb | 0 | --- | --- | --- |
| Site |  |  |  |  |
| URMC | -0.03 | -0.13 | 0.06 | 0.5100 |
| UNC | -0.01 | -0.11 | 0.09 | 0.7921 |
| UCSF | 0 | --- | --- | --- |
| GSTM1 status |  |  |  |  |
| Sufficient | 0.02 | -0.09 | 0.13 | 0.7073 |
| Null | 0 | --- | --- | --- |
| Ozone exposure by GSTM1 status |  |  |  |  |
| 120 ppb by sufficient | 0.04 | -0.09 | 0.18 | 0.5448 |
| 70 ppb by sufficient | 0.01 | -0.13 | 0.14 | 0.9403 |
| 0 ppb by sufficient | 0 | --- | --- | --- |
| 120 ppb by null | 0 | --- | --- | --- |
| 70 ppb by null | 0 | --- | --- | --- |
| 0 ppb by null | 0 | --- | --- | --- |

GSTM1, glutathione S-transferase Mu 1 gene; URMC, University of Rochester Medical Center; UNC, University of North Carolina; UCSF, University of California San Francisco.

**Table Mm. Type III sum of squares for change in endothelin-1 (pg/mL) including ozone exposure by time interaction.**

| Effect | P-value |
| --- | --- |
| 4 hr vs. 22 hr change | 0.2607 |
| Ozone exposure | 0.0082 |
| Site | 0.8427 |
| Ozone exposure by time | 0.9546 |

**Table Nn. Mixed model for change in endothelin-1 (pg/mL) including ozone exposure by time interaction.**

| Effect | Estimate | Lower  95% CI | Upper  95% CI | P-value |
| --- | --- | --- | --- | --- |
| Intercept | -0.01 | -0.10 | 0.08 | 0.8178 |
| 22 hr change | -0.02 | -0.11 | 0.07 | 0.6602 |
| 4 hr change | 0 | --- | --- | --- |
| Ozone exposure |  |  |  |  |
| 120 ppb | 0.08 | -0.01 | 0.17 | 0.0957 |
| 70 ppb | -0.02 | -0.11 | 0.08 | 0.7076 |
| 0 ppb | 0 | --- | --- | --- |
| Site |  |  |  |  |
| URMC | -0.03 | -0.12 | 0.07 | 0.5684 |
| UNC | 0 | -0.11 | 0.09 | 0.8421 |
| UCSF | 0 | --- | --- | --- |
| Ozone exposure by time |  |  |  |  |
| 120 ppb | -0.01 | -0.14 | 0.12 | 0.8854 |
| 70 ppb | -0.02 | -0.15 | 0.11 | 0.7611 |
| 0 ppb | 0 | --- | --- | --- |

URMC, University of Rochester Medical Center; UNC, University of North Carolina; UCSF, University of California San Francisco.

**Table Oo. Type III sum of squares for change in monocyte-platelet conjugate count including ozone concentration by age interaction.**

| **Effect** | **P-value** |
| --- | --- |
| Ozone concentration | 0.9081 |
| 4 h vs. 22 h change | 0.2569 |
| Site | 0.0792 |
| Age | 0.5372 |
| Ozone concentration by age | 0.0129 |

**Table Pp. Mixed model for change in monocyte-platelet conjugate count including ozone concentration by age interaction.**

| Effect | Estimate | Lower 95% CI | Upper 95% CI | P-value |
| --- | --- | --- | --- | --- |
| Intercept | -5.5 | -14.5 | 3.5 | 0.2277 |
| 4 h change | 3.1 | -2.3 | 8.4 | 0.2569 |
| 22 h change | 0 | --- | --- | --- |
| Ozone exposure |  |  |  |  |
| 120 ppb | 0.0 | -6.6 | 6.5 | 0.9956 |
| 70 ppb | -1.3 | -7.9 | 5.3 | 0.7012 |
| 0 ppb | 0 | --- | --- | --- |
| Site |  |  |  |  |
| URMC | 7.4 | -3.0 | 17.9 | 0.1604 |
| UNC | -5.0 | -16.5 | 6.5 | 0.3875 |
| UCSF | 0 | --- | --- | --- |
| Age | 0.9 | -0.4 | 2.2 | 0.1721 |
| Ozone exposure by age |  |  |  |  |
| 120 ppb by age | -1.5 | -3.0 | -0.1 | 0.0413 |
| 70 ppb by age | -2.2 | -3.6 | -0.7 | 0.0041 |
| 0 ppb by age | 0 | --- | --- | --- |

URMC, University of Rochester Medical Center; UNC, University of North Carolina; UCSF, University of California San Francisco.

**Table Qq. Type III sum of squares for change in monocyte-platelet conjugate count including ozone concentration by sex interaction.**

| Effect | P-value |
| --- | --- |
| Ozone concentration | 0.7431 |
| 4 h vs. 22 h change | 0.2610 |
| Site | 0.0932 |
| Sex | 0.2788 |
| Ozone concentration by sex | 0.0565 |

**Table Rr. Mixed model for change in monocyte-platelet conjugate count including ozone concentration by sex interaction.**

| Effect | Estimate | Lower 95% CI | Upper 95% CI | P-value |
| --- | --- | --- | --- | --- |
| Intercept | -1.8 | -12.7 | 9.1 | 0.7423 |
| 4 h change | 3.1 | -2.3 | 8.4 | 0.2610 |
| 22 h change | 0 | --- | --- | --- |
| Ozone exposure |  |  |  |  |
| 120 ppb | -8.6 | -18.7 | 1.5 | 0.0951 |
| 70 ppb | -9.5 | -19.6 | 0.6 | 0.0661 |
| 0 ppb | 0 | --- | --- | --- |
| Site |  |  |  |  |
| URMC | 5.8 | -4.5 | 16.2 | 0.2654 |
| UNC | -6.1 | -17.6 | 5.3 | 0.2907 |
| UCSF | 0 | --- | --- | --- |
| Sex |  |  |  |  |
| Female | -4.5 | -16.3 | 7.3 | 0.4484 |
| Male | 0 |  |  |  |
| Ozone exposure by sex |  | --- | --- | --- |
| 120 ppb by female | 14.5 | 1.2 | 27.8 | 0.0328 |
| 70 ppb by female | 13.7 | 0.4 | 27.1 | 0.0443 |
| 0 ppb by female | 0 | --- | --- | --- |
| 120 ppb by male | 0 | --- | --- | --- |
| 70 ppb by male | 0 | --- | --- | --- |
| 0 ppb by male | 0 | --- | --- | --- |

URMC, University of Rochester Medical Center; UNC, University of North Carolina; UCSF, University of California San Francisco.

**Table Ss. Type III sum of squares for change in monocyte-platelet conjugate count including ozone concentration by GSTM1 status interaction.**

| Effect | P-value |
| --- | --- |
| Ozone concentration | 0.9390 |
| 4 h vs. 22 h change | 0.2556 |
| Site | 0.0928 |
| GSTM1 status | 0.5940 |
| Ozone concentration by GSTM1 status | 0.0488 |

GSTM1, glutathione S-transferase Mu 1 gene.

**Table Tt. Mixed model for change in monocyte-platelet conjugate count including ozone concentration by GSTM1 status interaction.**

| Effect | Estimate | Lower 95% CI | Upper 95% CI | P-value |
| --- | --- | --- | --- | --- |
| Intercept | -2.2 | -12.1 | 7.8 | 0.6692 |
| 4 h change | 3.1 | -2.3 | 8.5 | 0.2556 |
| 22 h change | 0 | --- | --- | --- |
| Ozone exposure |  |  |  |  |
| 120 ppb | -5.0 | -13.6 | 3.5 | 0.2454 |
| 70 ppb | -8.4 | -17.1 | 0.2 | 0.0567 |
| 0 ppb | 0 | --- | --- | --- |
| Site |  |  |  |  |
| URMC | 6.6 | -3.7 | 17.0 | 0.2064 |
| UNC | -5.3 | -16.7 | 6.1 | 0.3598 |
| UCSF | 0 | --- | --- | --- |
| GSTM1 status |  |  |  |  |
| Sufficient | -6.9 | -18.7 | 4.8 | 0.2445 |
| Null | 0 | --- | --- | --- |
| Ozone exposure by GSTM1 status |  |  |  |  |
| 120 ppb by sufficient | 11.8 | -1.6 | 25.1 | 0.0841 |
| 70 ppb by sufficient | 16.3 | 2.9 | 29.6 | 0.0176 |
| 0 ppb by sufficient | 0 | --- | --- | --- |
| 120 ppb by null | 0 | --- | --- | --- |
| 70 ppb by null | 0 | --- | --- | --- |
| 0 ppb by null | 0 | --- | --- | --- |

GSTM1, glutathione S-transferase Mu 1 gene; URMC, University of Rochester Medical Center; UNC, University of North Carolina; UCSF, University of California San Francisco.

**Table Uu. Type III sum of squares for change in activated platelet count including ozone concentration by age interaction.**

| Effect | P-value |
| --- | --- |
| Ozone concentration | 0.7647 |
| 4 h vs. 22 h change | 0.5134 |
| Site | 0.0614 |
| Age | 0.4694 |
| Ozone concentration by age | 0.0457 |

**Table Vv. Mixed model for change in activated platelet count including ozone concentration by age interaction.**

| Effect | Estimate | Lower  95% CI | Upper  95% CI | P-value |
| --- | --- | --- | --- | --- |
| Intercept | -4995.5 | -9693.9 | -297.0 | 0.0375 |
| 4 h change | -1146.6 | -4620.6 | 2327.4 | 0.5134 |
| 22 h change | 0 | --- | --- | --- |
| Ozone exposure |  |  |  |  |
| 120 ppb | -1395.7 | -5625.2 | 2833.8 | 0.5155 |
| 70 ppb | -75.7 | -4337.7 | 4186.4 | 0.9721 |
| 0 ppb | 0 | --- | --- | --- |
| Site |  |  |  |  |
| URMC | 5372.6 | 469.9 | 10275.0 | 0.0321 |
| UNC | 5185.0 | 12.0 | 10358.0 | 0.0495 |
| UCSF | 0 | --- | --- | --- |
| Age | 799.5 | 85.9 | 1513.1 | 0.0286 |
| Ozone concentration by age |  |  |  |  |
| 120 ppb by age | -715.7 | -1645.1 | 213.7 | 0.1308 |
| 70 ppb by age | -1186.8 | -2129.4 | -244.2 | 0.0137 |
| 0 ppb by age | 0 | --- | --- | --- |

URMC, University of Rochester Medical Center; UNC, University of North Carolina; UCSF, University of California San Francisco.

**Table Ww. Type III sum of squares for change in activated platelet count including ozone concentration by sex interaction.**

| Effect | P-value |
| --- | --- |
| Ozone concentration | 0.7095 |
| 4 h vs. 22 h change | 0.5018 |
| Site | 0.0784 |
| Sex | 0.7670 |
| Ozone concentration by sex | 0.7642 |

**Table Xx. Mixed model for change in activated platelet count including ozone concentration by sex interaction.**

| Effect | Estimate | Lower  95% CI | Upper  95% CI | P-value |
| --- | --- | --- | --- | --- |
| Intercept | -4672.2 | -10584.0 | 1239.9 | 0.1198 |
| 4 h change | -1186.6 | -4684.5 | 2311.4 | 0.5018 |
| 22 h change | 0 | --- | --- | --- |
| Ozone exposure |  |  |  |  |
| 120 ppb | -3047.7 | -9756.5 | 3661.1 | 0.3709 |
| 70 ppb | -193.3 | -6902.2 | 6515.7 | 0.9547 |
| 0 ppb | 0 | --- | --- | --- |
| Site |  |  |  |  |
| URMC | 5415.6 | 474.0 | 10357.0 | 0.0321 |
| UNC | 4709.5 | -597.7 | 10017.0 | 0.0812 |
| UCSF | 0 | --- | --- | --- |
| Sex |  |  |  |  |
| Female | -194.8 | -6782.2 | 6392.6 | 0.9532 |
| Male | 0 | --- | --- | --- |
| Ozone concentration by sex |  |  |  |  |
| 120 ppb by female | 2686.9 | -5990.0 | 11364.0 | 0.5416 |
| 70 ppb by female | -202.1 | -8916.5 | 8512.4 | 0.9635 |
| 0 ppb by female | 0 | --- | --- | --- |
| 120 ppb by male | 0 | --- | --- | --- |
| 70 ppb by male | 0 | --- | --- | --- |
| 0 ppb by male | 0 | --- | --- | --- |

URMC, University of Rochester Medical Center; UNC, University of North Carolina; UCSF, University of California San Francisco.

**Table Yy. Type III sum of squares for change in activated platelet count including ozone concentration by GSTM1 status interaction.**

| Effect | P-value |
| --- | --- |
| Ozone concentration | 0.8851 |
| 4 h vs. 22 h change | 0.5060 |
| Site | 0.0566 |
| GSTM1 status | 0.6518 |
| Ozone concentration by GSTM1 status | 0.0732 |

GSTM1, glutathione S-transferase Mu 1 gene.

**Table Zz. Mixed model for change in activated platelet count including ozone concentration by GSTM1 status interaction.**

| Effect | Estimate | Lower  95% CI | Upper  95% CI | P-value |
| --- | --- | --- | --- | --- |
| Intercept | -2142.5 | -7468.7 | 3183.8 | 0.4259 |
| 4r change | -1169 | -4649.5 | 2311.4 | 0.5060 |
| 22 h change | 0 | --- | --- | --- |
| Ozone exposure |  |  |  |  |
| 120 ppb | -5018.1 | -10535.0 | 499.0 | 0.0743 |
| 70 ppb | -3996.7 | -9614.2 | 1620.8 | 0.1619 |
| 0 ppb | 0 | --- | --- | --- |
| Site |  |  |  |  |
| URMC | 5667.1 | 777.4 | 10557.0 | 0.0237 |
| UNC | 4904.3 | -265.8 | 10074.0 | 0.0627 |
| UCSF | 0 | --- | --- | --- |
| GSTM1 status |  |  |  |  |
| Sufficient | -6745.8 | -13243.0 | -248.3 | 0.0421 |
| Null | 0 | --- | --- | --- |
| Ozone concentration by GSTM1 status |  |  |  |  |
| 120 ppb by sufficient | 8672.7 | 70.0 | 17275.0 | 0.0482 |
| 70 ppb by sufficient | 8752.8 | 131.3 | 17374.0 | 0.0467 |
| 0 ppb by sufficient | 0 | --- | --- | --- |
| 120 ppb by null | 0 | --- | --- | --- |
| 70 ppb by null | 0 | --- | --- | --- |
| 0 ppb by null | 0 | --- | --- | --- |

GSTM1, glutathione S-transferase Mu 1 gene; URMC, University of Rochester Medical Center; UNC, University of North Carolina; UCSF, University of California San Francisco.

**Table Aaa. Type III sum of squares for change in MP-TFA (pg/mL) including ozone concentration by age interaction.**

| Effect | P-value |
| --- | --- |
| Ozone concentration | 0.8289 |
| 4 h vs. 22 h change | 0.5092 |
| Site | 0.3463 |
| Age | 0.0939 |
| Ozone concentration by age | 0.1326 |

MP-TFA, microparticle-associated tissue factor activity.

**Table Bbb. Mixed model for change in MP-TFA (pg/mL) including ozone concentration by age interaction.**

| Effect | Estimate | Lower  95% CI | Upper  95% CI | P-value |
| --- | --- | --- | --- | --- |
| Intercept | 0.019 | -0.029 | 0.067 | 0.4365 |
| 4 hr change | 0.011 | -0.021 | 0.043 | 0.5092 |
| 22 hr change | 0 | --- | --- | --- |
| Ozone exposure |  |  |  |  |
| 120 ppb | 0.008 | -0.031 | 0.047 | 0.6826 |
| 70 ppb | -0.004 | -0.043 | 0.036 | 0.8534 |
| 0 ppb | 0 | --- | --- | --- |
| Site |  |  |  |  |
| URMC | -0.027 | -0.08 | 0.026 | 0.3086 |
| UNC | -0.039 | -0.093 | 0.015 | 0.1562 |
| UCSF | 0 | --- | --- | --- |
| Age | 0.005 | -0.002 | 0.012 | 0.1441 |
| Ozone exposure by age |  |  |  |  |
| 120 ppb by age | 0.003 | -0.006 | 0.011 | 0.5466 |
| 70 ppb by age | -0.006 | -0.014 | 0.003 | 0.1774 |
| 0 ppb by age | 0 | --- | --- | --- |

MP-TFA, microparticle-associated tissue factor activity; URMC, University of Rochester Medical Center; UNC, University of North Carolina; UCSF, University of California San Francisco.

**Table Ccc. Type III sum of squares for change in MP-TFA (pg/mL) including ozone concentration by sex interaction.**

| Effect | P-value |
| --- | --- |
| Ozone concentration | 0.8791 |
| 4 hr vs. 22 hr change | 0.5015 |
| Site | 0.5611 |
| Sex | 0.0233 |
| Ozone concentration by sex | 0.1426 |

MP-TFA, microparticle-associated tissue factor activity.

**Table Ddd. Mixed model for change in MP-TFA (pg/mL) including ozone concentration by sex interaction.**

| Effect | Estimate | Lower  95% CI | Upper  95% CI | P-value |
| --- | --- | --- | --- | --- |
| Intercept | 0.029 | -0.029 | 0.087 | 0.3237 |
| 4 hr change | 0.011 | -0.021 | 0.043 | 0.5015 |
| 22 hr change | 0 | --- | --- | --- |
| Ozone exposure |  |  |  |  |
| 120 ppb | 0.008 | -0.052 | 0.068 | 0.7912 |
| 70 ppb | 0.035 | -0.025 | 0.095 | 0.2565 |
| 0 ppb | 0 | --- | --- | --- |
| Site |  |  |  |  |
| URMC | -0.014 | -0.067 | 0.038 | 0.5938 |
| UNC | -0.03 | -0.084 | 0.025 | 0.2845 |
| UCSF | 0 | --- | --- | --- |
| Sex |  |  |  |  |
| Female | -0.029 | -0.093 | 0.035 | 0.3661 |
| Male | 0 | --- | --- | --- |
| Ozone exposure by sex |  |  |  |  |
| 120 ppb by female | 0.002 | -0.077 | 0.081 | 0.9555 |
| 70 ppb by female | -0.068 | -0.147 | 0.012 | 0.0941 |
| 0 ppb by female | 0 | --- | --- | --- |
| 120 ppb by male | 0 | --- | --- | --- |
| 70 ppb by male | 0 | --- | --- | --- |
| 0 ppb by male | 0 | --- | --- | --- |

MP-TFA, microparticle-associated tissue factor activity; URMC, University of Rochester Medical Center; UNC, University of North Carolina; UCSF, University of California San Francisco.

**Table Eee. Type III sum of squares for change in MP-TFA (pg/mL) including ozone concentration by GSTM1 status interaction.**

| Effect | P-value |
| --- | --- |
| Ozone concentration | 0.5484 |
| 4 h vs. 22 h change | 0.5040 |
| Site | 0.3016 |
| GSTM1 status | 0.3088 |
| Ozone concentration by GSTM1 status | 0.0300 |

MP-TFA, microparticle-associated tissue factor activity; GSTM1, glutathione S-transferase Mu 1 gene.

**Table Fff. Mixed model for change in MP-TFA (pg/mL) including ozone concentration by GSTM1 status interaction.**

| Effect | Estimate | Lower  95% CI | Upper  95% CI | P-value |
| --- | --- | --- | --- | --- |
| Intercept | 0.028 | -0.026 | 0.082 | 0.3050 |
| 4 h change | 0.011 | -0.021 | 0.043 | 0.5040 |
| 22 h change | 0 | --- | --- | --- |
| Ozone exposure |  |  |  |  |
| 120 ppb | -0.034 | -0.084 | 0.017 | 0.1917 |
| 70 ppb | -0.015 | -0.066 | 0.035 | 0.5498 |
| 0 ppb | 0 | --- | --- | --- |
| Site |  |  |  |  |
| URMC | -0.025 | -0.078 | 0.029 | 0.3591 |
| UNC | -0.043 | -0.098 | 0.012 | 0.1235 |
| UCSF | 0 | --- | --- | --- |
| GSTM1 status |  |  |  |  |
| Sufficient | -0.020 | -0.085 | 0.045 | 0.5391 |
| Null | 0 | --- | --- | --- |
| Ozone exposure by GSTM1 status |  |  |  |  |
| 120 ppb by sufficient | 0.103 | 0.024 | 0.182 | 0.0112 |
| 70 ppb by sufficient | 0.026 | -0.053 | 0.106 | 0.5176 |
| 0 ppb by sufficient | 0 | --- | --- | --- |
| 120 ppb by null | 0 | --- | --- | --- |
| 70 ppb by null | 0 | --- | --- | --- |
| 0 ppb by null | 0 | --- | --- | --- |

MP-TFA, microparticle-associated tissue factor activity; GSTM1, glutathione S-transferase Mu 1 gene; URMC, University of Rochester Medical Center; UNC, University of North Carolina; UCSF, University of California San Francisco.

**Table Ggg. Type III sum of squares for change in platelet MP count including ozone concentration by age interaction.**

| Effect | P-value |
| --- | --- |
| Ozone concentration | 0.5122 |
| 4 h vs. 22 h change | 0.2762 |
| Site | 0.4120 |
| Age | 0.2189 |
| Ozone concentration by age | 0.2217 |

MP, microparticle.

**Table Hhh. Mixed model for change in platelet MP count including ozone concentration by age interaction.**

| Effect | Estimate | Lower  95% CI | Upper  95% CI | P-value |
| --- | --- | --- | --- | --- |
| Intercept | -1113.1 | -1820.2 | -406.0 | 0.0024 |
| 4 h change | 268.8 | -218.9 | 756.4 | 0.2762 |
| 22 h change | 0 | --- | --- | --- |
| Ozone exposure |  |  |  |  |
| 120 ppb | 196.2 | -399.3 | 791.7 | 0.5162 |
| 70 ppb | 350.5 | -248.3 | 949.2 | 0.2493 |
| 0 ppb | 0 | --- | --- | --- |
| Site |  |  |  |  |
| URMC | 500.1 | -263.7 | 1264.0 | 0.1964 |
| UNC | 382.1 | -419.1 | 1183.2 | 0.3455 |
| UCSF | 0 | --- | --- | --- |
| Age | 26.4 | -78.6 | 131.4 | 0.6182 |
| Ozone concentration by age |  |  |  |  |
| 120 ppb by age | 81.7 | -49.4 | 212.9 | 0.2210 |
| 70 ppb by age | -29.8 | -162.4 | 102.8 | 0.6586 |
| 0 ppb by age | 0 | --- | --- | --- |

MP, microparticle; URMC, University of Rochester Medical Center; UNC, University of North Carolina; UCSF, University of California San Francisco.

**Table Iii. Type III sum of squares for change in platelet MP count including ozone concentration by sex interaction**

| Effect | P-value |
| --- | --- |
| Ozone concentration | 0.4374 |
| 4 h vs. 22 h change | 0.2759 |
| Site | 0.3231 |
| Sex | 0.6830 |
| Ozone concentration by sex | 0.4511 |

MP, microparticle.

**Table Jjj. Mixed model for change in platelet MP count including ozone concentration by sex interaction**

| Effect | Estimate | Lower  95% CI | Upper  95% CI | P-value |
| --- | --- | --- | --- | --- |
| Intercept | -1299.0 | -2184.6 | -413.4 | 0.0045 |
| 4 h change | 269.5 | -219.1 | 758.1 | 0.2759 |
| 22 h change | 0 | --- | --- | --- |
| Ozone exposure |  |  |  |  |
| 120 ppb | 687.0 | -262.4 | 1636.4 | 0.1549 |
| 70 ppb | 571.1 | -372.3 | 1514.4 | 0.2335 |
| 0 ppb | 0 | --- | --- | --- |
| Site |  |  |  |  |
| URMC | 579.7 | -191.3 | 1350.6 | 0.1385 |
| UNC | 415.7 | -407.7 | 1239.2 | 0.3182 |
| UCSF | 0 | --- | --- | --- |
| Sex |  |  |  |  |
| Female | 252.9 | -715.8 | 1221.6 | 0.6049 |
| Male | 0 | --- | --- | --- |
| Ozone concentration by sex |  |  |  |  |
| 120 ppb by female | -781.4 | -2001.7 | 438.9 | 0.2078 |
| 70 ppb by female | -384.3 | -1605.3 | 836.8 | 0.5350 |
| 0 ppb by female | 0 | --- | --- | --- |
| 120 ppb by male | 0 | --- | --- | --- |
| 70 ppb by male | 0 | --- | --- | --- |
| 0 ppb by male | 0 | --- | --- | --- |

MP, microparticle; URMC, University of Rochester Medical Center; UNC, University of North Carolina; UCSF, University of California San Francisco.

**Table Kkk. Type III sum of squares for change in platelet MP count including ozone concentration by GSTM1 status interaction.**

| Effect | P-value |
| --- | --- |
| Ozone concentration | 0.6542 |
| 4 h vs. 22 h change | 0.2768 |
| Site | 0.3157 |
| GSTM1 status | 0.4819 |
| Ozone concentration by GSTM1 status | 0.4145 |

MP, microparticle; GSTM1, glutathione S-transferase Mu 1 gene.

**Table Lll. Mixed model for change in platelet MP count including ozone concentration by GSTM1 status interaction.**

| Effect | Estimate | Lower  95% CI | Upper  95% CI | P-value |
| --- | --- | --- | --- | --- |
| Intercept | -1242.4 | -2035.7 | -449.0 | 0.0025 |
| 4 h change | 269.0 | -219.7 | 757.6 | 0.2768 |
| 22 h change | 0 | --- | --- | --- |
| Ozone exposure |  |  |  |  |
| 120 ppb | 472.7 | -299.7 | 1245.1 | 0.2285 |
| 70 ppb | 663.6 | -123.7 | 1451.0 | 0.0979 |
| 0 ppb | 0 | --- | --- | --- |
| Site |  |  |  |  |
| URMC | 583.8 | -183.0 | 1350.5 | 0.1337 |
| UNC | 400.3 | -406.2 | 1206.7 | 0.3264 |
| UCSF | 0 | --- | --- | --- |
| GSTM1 status |  |  |  |  |
| Sufficient | 239.3 | -720.6 | 1199.2 | 0.6212 |
| Null | 0 | --- | --- | --- |
| Ozone concentration by GSTM1 status |  |  |  |  |
| 120 ppb by sufficient | -642.3 | -1858.1 | 573.4 | 0.2982 |
| 70 ppb by sufficient | -761.1 | -1974.5 | 452.4 | 0.2172 |
| 0 ppb by sufficient | 0 | --- | --- | --- |
| 120 ppb by null | 0 | --- | --- | --- |
| 70 ppb by null | 0 | --- | --- | --- |
| 0 ppb by null | 0 | --- | --- | --- |

MP, microparticle; GSTM1, glutathione S-transferase Mu 1 gene; URMC, University of Rochester Medical Center; UNC, University of North Carolina; UCSF, University of California San Francisco.**Table Mmm. Type III sum of squares for change in activated platelet MP count including ozone concentration by age interaction.**

| Effect | P-value |
| --- | --- |
| Ozone concentration | 0.5189 |
| 4 h vs. 22 h change | 0.3948 |
| Site | 0.2621 |
| Age | 0.1497 |
| Ozone concentration by age | 0.4373 |

MP, microparticle.

**Table Nnn. Mixed model for change in activated platelet MP count including ozone concentration by age interaction.**

| Effect | Estimate | Lower  95% CI | Upper  95% CI | P-value |
| --- | --- | --- | --- | --- |
| Intercept | -281.6 | -490.3 | -72.9 | 0.0088 |
| 4 h change | 59.1 | -78.3 | 196.5 | 0.3948 |
| 22 h change | 0 | --- | --- | --- |
| Ozone exposure |  |  |  |  |
| 120 ppb | 74.1 | -93.8 | 242.1 | 0.3846 |
| 70 ppb | 92.6 | -76.3 | 261.5 | 0.2806 |
| 0 ppb | 0 | --- | --- | --- |
| Site |  |  |  |  |
| URMC | 181.9 | -49.1 | 412.8 | 0.1210 |
| UNC | 46.9 | -194.8 | 288.5 | 0.7005 |
| UCSF | 0 | --- | --- | --- |
| Age | 29.5 | -1.1 | 60.0 | 0.0589 |
| Ozone concentration by age |  |  |  |  |
| 120 ppb by age | -19.1 | -56.0 | 17.9 | 0.3114 |
| 70 ppb by age | -22.8 | -60.2 | 14.6 | 0.2316 |
| 0 ppb by age | 0 | --- | --- | --- |

MP, microparticle; URMC, University of Rochester Medical Center; UNC, University of North Carolina; UCSF, University of California San Francisco.

**Table Ooo. Type III sum of squares for change in activated platelet MP count including ozone concentration by sex interaction.**

| **Effect** | **P-value** |
| --- | --- |
| Ozone concentration | 0.4616 |
| 4 h vs. 22 h change | 0.3987 |
| Site | 0.1574 |
| Sex | 0.3650 |
| Ozone concentration by sex | 0.8387 |

MP, microparticle.

**Table Ppp. Mixed model for change in activated platelet MP count including ozone concentration by sex interaction.**

| Effect | Estimate | Lower  95% CI | Upper  95% CI | P-value |
| --- | --- | --- | --- | --- |
| Intercept | -278.5 | -537.5 | -19.5 | 0.0354 |
| 4 h change | 58.8 | -79.0 | 196.5 | 0.3987 |
| 22 h change | 0 | --- | --- | --- |
| Ozone exposure |  |  |  |  |
| 120 ppb | 116.4 | -151.4 | 384.2 | 0.3918 |
| 70 ppb | 153.3 | -112.9 | 419.5 | 0.2569 |
| 0 ppb | 0 | --- | --- | --- |
| Site |  |  |  |  |
| URMC | 217.3 | -14.9 | 449.6 | 0.0663 |
| UNC | 66.6 | -180.9 | 314.1 | 0.5938 |
| UCSF | 0 | --- | --- | --- |
| Sex |  |  |  |  |
| Female | -34.3 | -316.2 | 247.6 | 0.8094 |
| Male | 0 | --- | --- | --- |
| Ozone concentration by sex |  |  |  |  |
| 120 ppb by female | -67.8 | -412.1 | 276.4 | 0.6977 |
| 70 ppb by female | -101.6 | -446.2 | 242.9 | 0.5610 |
| 0 ppb by female | 0 | --- | --- | --- |
| 120 ppb by male | 0 | --- | --- | --- |
| 70 ppb by male | 0 | --- | --- | --- |
| 0 ppb by male | 0 | --- | --- | --- |

MP, microparticle; URMC, University of Rochester Medical Center; UNC, University of North Carolina; UCSF, University of California San Francisco.

**Table Qqq. Type III sum of squares for change in activated platelet MP count including ozone concentration by GSTM1 status interaction.**

| Effect | P-value |
| --- | --- |
| Ozone concentration | 0.5229 |
| 4 h vs. 22 h change | 0.3992 |
| Site | 0.1594 |
| GSTM1 status | 0.3897 |
| Ozone concentration by GSTM1 status | 0.6417 |

MP, microparticle; GSTM1, glutathione S-transferase Mu 1 gene.

**Table Rrr. Mixed model for change in activated platelet MP count including ozone concentration by GSTM1 status interaction.**

| Effect | Estimate | Lower  95% CI | Upper  95% CI | P-value |
| --- | --- | --- | --- | --- |
| Intercept | -258.3 | -491.7 | -25 | 0.0305 |
| 4 h change | 58.7 | -79.0 | 196.3 | 0.3992 |
| 22 h change | 0 | --- | --- | --- |
| Ozone exposure |  |  |  |  |
| 120 ppb | 43.8 | -173.9 | 261.5 | 0.6914 |
| 70 ppb | 130.9 | -91.2 | 352.9 | 0.2461 |
| 0 ppb | 0 | --- | --- | --- |
| Site |  |  |  |  |
| URMC | 212.2 | -19.8 | 444.2 | 0.0725 |
| UNC | 49.9 | -193.5 | 293.3 | 0.6845 |
| UCSF | 0 | --- | --- | --- |
| GSTM1 status |  |  |  |  |
| Sufficient | -82.2 | -361.7 | 197.4 | 0.5604 |
| Null | 0 | --- | --- | --- |
| Ozone concentration by GSTM1 status |  |  |  |  |
| 120 ppb by sufficient | 77.9 | -264.8 | 420.6 | 0.6539 |
| 70 ppb by sufficient | -85.0 | -427.0 | 257.1 | 0.6244 |
| 0 ppb by sufficient | 0 | --- | --- | --- |
| 120 ppb by null | 0 | --- | --- | --- |
| 70 ppb by null | 0 | --- | --- | --- |
| 0 ppb by null | 0 | --- | --- | --- |

MP, microparticle; GSTM1, glutathione S-transferase Mu 1 gene; URMC, University of Rochester Medical Center; UNC, University of North Carolina; UCSF, University of California San Francisco.

**Table Sss. Type III sum of squares for change in CD142+ MP count including ozone concentration by age interaction.**

| Effect | P-value |
| --- | --- |
| Ozone concentration | 0.5787 |
| 4 h vs. 22 h change | 0.5985 |
| Site | 0.1251 |
| Age | 0.7937 |
| Ozone concentration by age | 0.3956 |

MP, microparticle.

**Table Ttt. Mixed model for change in CD142+ MP count including ozone concentration by age interaction.**

| Effect | Estimate | Lower  95% CI | Upper  95% CI | P-value |
| --- | --- | --- | --- | --- |
| Intercept | -2095.6 | -12465.0 | 8273.9 | 0.6887 |
| 4 h change | 1842.8 | -5086.5 | 8770.0 | 0.5985 |
| 22 h change | 0 | --- | --- | --- |
| Ozone exposure |  |  |  |  |
| 120 ppb | -4369.0 | -12864.0 | 4125.7 | 0.3112 |
| 70 ppb | -1250.4 | -9762.1 | 7261.2 | 0.7720 |
| 0 ppb | 0 | --- | --- | --- |
| Site |  |  |  |  |
| URMC | -2092.1 | -13504.0 | 9320.1 | 0.7163 |
| UNC | -11510.0 | -23427.0 | 408.0 | 0.0582 |
| UCSF | 0 | --- | --- | --- |
| Age | 95.9 | -1433.0 | 1624.9 | 0.9010 |
| Ozone concentration by age |  |  |  |  |
| 120 ppb by age | -580.2 | -2445.8 | 1285.4 | 0.5413 |
| 70 ppb by age | 706.2 | -1177.3 | 2589.8 | 0.4614 |
| 0 ppb by age | 0 | --- | --- | --- |

MP, microparticle; URMC, University of Rochester Medical Center; UNC, University of North Carolina; UCSF, University of California San Francisco.

**Table Uuu. Type III sum of squares for change in CD142+ MP count including ozone concentration by sex interaction.**

| Effect | P-value |
| --- | --- |
| Ozone concentration | 0.9085 |
| 4 h vs. 22 h change | 0.5869 |
| Site | 0.0887 |
| Sex | 0.2403 |
| Ozone concentration by sex | 0.0231 |

MP, microparticle.

**Table Vvv. Mixed model for change in CD142+ MP count including ozone concentration by sex interaction.**

| Effect | Estimate | Lower  95% CI | Upper  95% CI | P-value |
| --- | --- | --- | --- | --- |
| Intercept | -10472.0 | -23309.0 | 2365.4 | 0.1085 |
| 4 h change | 1885.1 | -4987.9 | 8758.2 | 0.5869 |
| 22 h change | 0 | --- | --- | --- |
| Ozone exposure |  |  |  |  |
| 120 ppb | 9699. 9 | -3868.3 | 23268.0 | 0.1599 |
| 70 ppb | 1989.9 | -11365.0 | 15345.0 | 0.7689 |
| 0 ppb | 0 | --- | --- | --- |
| Site |  |  |  |  |
| URMC | -2925.0 | -14296.0 | 8445.8 | 0.6102 |
| UNC | -12787.0 | -24867.0 | -707.5 | 0.0383 |
| UCSF | 0 | --- | --- | --- |
| Sex |  |  |  |  |
| Female | 15002.0 | 994.4 | 29009.0 | 0.0361 |
| Male | 0 | --- | --- | --- |
| Ozone concentration by sex |  |  |  |  |
| 120 ppb by female | -23011.0 | -40320.0 | -5702.3 | 0.0095 |
| 70 ppb by female | -4668.8 | -21886.0 | 12548.0 | 0.5929 |
| 0 ppb by female | 0 | --- | --- | --- |
| 120 ppb by male | 0 | --- | --- | --- |
| 70 ppb by male | 0 | --- | --- | --- |
| 0 ppb by male | 0 | --- | --- | --- |

MP, microparticle; URMC, University of Rochester Medical Center; UNC, University of North Carolina; UCSF, University of California San Francisco.

**Table Www. Type III sum of squares for change in CD142+ MP count including ozone concentration by GSTM1 status interaction.**

| Effect | P-value |
| --- | --- |
| Ozone concentration | 0.3831 |
| 4 h vs. 22 h change | 0.5965 |
| Site | 0.1155 |
| GSTM1 status | 0.2242 |
| Ozone concentration by GSTM1 status | 0.2470 |

MP, microparticle; GSTM1, glutathione S-transferase Mu 1 gene.

**Table Xxx. Mixed model for change in CD142+ MP count including ozone concentration by GSTM1 status interaction.**

| Effect | Estimate | Lower  95% CI | Upper  95% CI | P-value |
| --- | --- | --- | --- | --- |
| Intercept | -6579.7 | -18079.0 | 4919.6 | 0.2583 |
| 4 h change | 1848.1 | -5066.6 | 8762.7 | 0.5965 |
| 22 h change | 0 | --- | --- | --- |
| Ozone exposure |  |  |  |  |
| 120 ppb | 1257.6 | -9701.0 | 12216.0 | 0.8209 |
| 70 ppb | 457.5 | -10648.0 | 11563.0 | 0.9352 |
| 0 ppb | 0 | --- | --- | --- |
| Site |  |  |  |  |
| URMC | -2581.4 | -13927.0 | 8764.0 | 0.6520 |
| UNC | -11835.0 | -23714.0 | 44.3 | 0.0508 |
| UCSF | 0 | --- | --- | --- |
| GSTM1 status |  |  |  |  |
| Sufficient | 11894.0 | -2032.9 | 25821.0 | 0.0931 |
| Null | 0 | --- | --- | --- |
| Ozone concentration by GSTM1 status |  |  |  |  |
| 120 ppb by sufficient | -14198.0 | -31481.0 | 3085.7 | 0.1067 |
| 70 ppb by sufficient | -3922.2 | -21128.0 | 13284.0 | 0.6531 |
| 0 ppb by sufficient | 0 | --- | --- | --- |
| 120 ppb by null | 0 | --- | --- | --- |
| 70 ppb by null | 0 | --- | --- | --- |
| 0 ppb by null | 0 | --- | --- | --- |

MP, microparticle; GSTM1, glutathione S-transferase Mu 1 gene; URMC, University of Rochester Medical Center; UNC, University of North Carolina; UCSF, University of California San Francisco.

**Table Yyy. Type III sum of squares for change in CD40 ligand+ MP count including ozone concentration by age interaction.**

| Effect | P-value |
| --- | --- |
| Ozone concentration | 0.2953 |
| 4 h vs. 22 h change | 0.6412 |
| Site | 0.1209 |
| Age | 0.6063 |
| Ozone concentration by age | 0.3330 |

MP, microparticle.

**Table Zzz. Mixed model for change in CD40 ligand+ MP count including ozone concentration by age interaction.**

| Effect | Estimate | Lower  95% CI | Upper  95% CI | P-value |
| --- | --- | --- | --- | --- |
| Intercept | 45.5 | -12286.0 | 12377.0 | 0.9942 |
| 4 h change | 1682.41 | -5471.14 | 8836.0 | 0.6412 |
| 22 h change | 0 | --- | --- | --- |
| Ozone exposure |  |  |  |  |
| 120 ppb | -6442.2 | -15236.0 | 2351.56 | 0.1499 |
| 70 ppb | -5573.8 | -14387.0 | 3239.3 | 0.2134 |
| 0 ppb | 0 | --- | --- | --- |
| Site |  |  |  |  |
| URMC | -3884.12 | -18272.0 | 10504.0 | 0.5927 |
| UNC | -14973.0 | -29922.0 | -24.3 | 0.0496 |
| UCSF | 0 | --- | --- | --- |
| Age | 14.6 | -1736.9 | 1766.0 | 0.9869 |
| Ozone concentration by age |  |  |  |  |
| 120 ppb by age | -181.6 | -2112.4 | 1749.2 | 0.8534 |
| 70 ppb by age | 1164.6 | -786.4 | 3115.6 | 0.2413 |
| 0 ppb by age | 0 | --- | --- | --- |

MP, microparticle; URMC, University of Rochester Medical Center; UNC, University of North Carolina; UCSF, University of California San Francisco.

**Table Aaaa. Type III sum of squares for change in CD40 ligand+ MP count including ozone concentration by sex interaction.**

| Effect | P-value |
| --- | --- |
| Ozone concentration | 0.5599 |
| 4 h vs. 22 h change | 0.6218 |
| Site | 0.0919 |
| Sex | 0.3167 |
| Ozone concentration by sex | 0.0008 |

MP, microparticle.

**Table Bbbb. Mixed model for change in CD40 ligand+ MP count including ozone concentration by sex interaction.**

| Effect | Estimate | Lower  95% CI | Upper  95% CI | P-value |
| --- | --- | --- | --- | --- |
| Intercept | -9788.9 | -24744.0 | 5166.2 | 0.1965 |
| 4 h change | 1750.8 | -5280.7 | 8782.3 | 0.6218 |
| 22 h change | 0 | --- | --- | --- |
| Ozone exposure |  |  |  |  |
| 120 ppb | 12844.0 | -1086.4 | 26774.0 | 0.0705 |
| 70 ppb | -3515.6 | -17232.0 | 10201.0 | 0.6133 |
| 0 ppb | 0 | --- | --- | --- |
| Site |  |  |  |  |
| URMC | -4601.2 | -19011.0 | 9808.9 | 0.5271 |
| UNC | -16293.0 | -31521.0 | -1065.5 | 0.0363 |
| UCSF | 0 | --- | --- | --- |
| Sex |  |  |  |  |
| Female | 17539.0 | 1478.9 | 33600.0 | 0.0327 |
| Male | 0 | --- | --- | --- |
| Ozone concentration by sex |  |  |  |  |
| 120 ppb by female | -31405.0 | -49167.0 | -13642.0 | 0.0006 |
| 70 ppb by female | -2572.1 | -20246.0 | 15102.0 | 0.7741 |
| 0 ppb by female | 0 | --- | --- | --- |
| 120 ppb by male | 0 | --- | --- | --- |
| 70 ppb by male | 0 | --- | --- | --- |
| 0 ppb by male | 0 | --- | --- | --- |

MP, microparticle; URMC, University of Rochester Medical Center; UNC, University of North Carolina; UCSF, University of California San Francisco.

**Table Cccc. Type III sum of squares for change in CD40 ligand+ MP count including ozone concentration by GSTM1 status interaction.**

| Effect | P-value |
| --- | --- |
| Ozone concentration | 0.2140 |
| 4 h vs. 22 h change | 0.6398 |
| Site | 0.1069 |
| GSTM1 status | 0.1435 |
| Ozone concentration by GSTM1 status | 0.4134 |

MP, microparticle; GSTM1, glutathione S-transferase Mu 1 gene.

**Table Dddd. Mixed model for change in CD40 ligand+ MP count including ozone concentration by GSTM1 status interaction.**

| Effect | Estimate | Lower  95% CI | Upper  95% CI | P-value |
| --- | --- | --- | --- | --- |
| Intercept | -5249.3 | -18770.0 | 8271.1 | 0.4421 |
| 4 h change | 1689.2 | -5463.2 | 8841.7 | 0.6398 |
| 22 h change | 0 | --- | --- | --- |
| Ozone exposure |  |  |  |  |
| 120 ppb | -1729.5 | -13092.0 | 9632.9 | 0.7640 |
| 70 ppb | -3604.4 | -15131.0 | 7922.1 | 0.5376 |
| 0 ppb | 0 | --- | --- | --- |
| Site |  |  |  |  |
| URMC | -4506.4 | -18767.0 | 9754.5 | 0.5313 |
| UNC | -15450.0 | -30309.0 | -591.3 | 0.0417 |
| UCSF | 0 | --- | --- | --- |
| GSTM1 status |  |  |  |  |
| Sufficient | 14277.0 | -1645.9 | 30200.0 | 0.0782 |
| Null | 0 | --- | --- | --- |
| Ozone concentration by GSTM1 status |  |  |  |  |
| 120 ppb by sufficient | -11938.0 | -29865.0 | 5990.0 | 0.1903 |
| 70 ppb by sufficient | -4357.7 | -22204.0 | 13489.0 | 0.6302 |
| 0 ppb by sufficient | 0 | --- | --- | --- |
| 120 ppb by null | 0 | --- | --- | --- |
| 70 ppb by null | 0 | --- | --- | --- |
| 0 ppb by null | 0 | --- | --- | --- |

MP, microparticle; GSTM1, glutathione S-transferase Mu 1 gene; URMC, University of Rochester Medical Center; UNC, University of North Carolina; UCSF, University of California San Francisco.

**Table Eeee. Type III sum of squares for change in platelet count (1000/uL) including ozone concentration by age interaction.**

| Effect | P-value |
| --- | --- |
| Ozone concentration | 0.1487 |
| 4hr v 22hr change | 0.0398 |
| Site | <.0001 |
| Age | 0.1343 |
| Ozone concentration by age | 0.1644 |

**Table Ffff. Mixed model for change in platelet count (1000/uL) including ozone concentration by age interaction.**

| Effect | Estimate | Lower  95% CI | Upper  95% CI | P-value |
| --- | --- | --- | --- | --- |
| Intercept | -14.6 | -19.0 | -10.2 | <.0001 |
| 4 h change | 2.6 | 0.1 | 5.1 | 0.0398 |
| 22 h change | 0 | --- | --- | --- |
| Ozone exposure |  |  |  |  |
| 120 ppb | 1.4 | -1.6 | 4.5 | 0.3533 |
| 70 ppb | -1.6 | -4.6 | 1.5 | 0.3103 |
| 0 ppb | 0 | --- | --- | --- |
| Site |  |  |  |  |
| URMC | 5.5 | 0.3 | 10.6 | 0.0382 |
| UNC | 13.5 | 8.2 | 18.8 | <.0001 |
| UCSF | 0 | --- | --- | --- |
| Age | -0.3 | -0.9 | 0.3 | 0.3279 |
| Ozone exposure by age |  |  |  |  |
| 120 ppb by age | -0.4 | -1.1 | 0.3 | 0.2395 |
| 70 ppb by age | 0.2 | -0.4 | 0.9 | 0.4841 |
| 0 ppb by age | 0 | --- | --- | --- |

URMC, University of Rochester Medical Center; UNC, University of North Carolina; UCSF, University of California San Francisco.

**Table Gggg. Type III sum of squares for change in platelet count (1000/uL) including ozone concentration by sex interaction.**

| Effect | P-value |
| --- | --- |
| Ozone concentration | 0.3896 |
| 4 h vs. 22 h change | 0.0398 |
| Site | <.0001 |
| Sex | 0.5119 |
| Ozone concentration by sex | 0.0259 |

**Table Hhhh. Mixed model for change in platelet count (1000/uL) including ozone concentration by sex interaction.**

| Effect | Estimate | Lower  95% CI | Upper  95% CI | P-value |
| --- | --- | --- | --- | --- |
| Intercept | -14.1 | -19.4 | -8.8 | <.0001 |
| 4 h change | 2.6 | 0.1 | 5.1 | 0.0398 |
| 22 h change | 0 | --- | --- | --- |
| Ozone exposure |  |  |  |  |
| 120 ppb | -0.8 | -5.4 | 3.9 | 0.7421 |
| 70 ppb | 1.3 | -3.3 | 5.9 | 0.5794 |
| 0 ppb | 0 | --- | --- | --- |
| Site |  |  |  |  |
| URMC | 5.3 | 0.0 | 10.5 | 0.0487 |
| UNC | 13.8 | 8.3 | 19.3 | <.0001 |
| UCSF | 0 | --- | --- | --- |
| Sex |  |  |  |  |
| Female | -1.1 | -6.8 | 4.6 | 0.6988 |
| Male | 0 | --- | --- | --- |
| Ozone exposure by sex |  |  |  |  |
| 120 ppb by female | 3.7 | -2.5 | 9.8 | 0.2394 |
| 70 ppb by female | -4.7 | -10.8 | 1.4 | 0.1282 |
| 0 ppb by female | 0 | --- | --- | --- |
| 120 ppb by male | 0 | --- | --- | --- |
| 70 ppb by male | 0 | --- | --- | --- |
| 0 ppb by male | 0 | --- | --- | --- |

URMC, University of Rochester Medical Center; UNC, University of North Carolina; UCSF, University of California San Francisco.

**Table Iiii. Type III sum of squares for change in platelet count (1000/uL) including ozone concentration by GSTM1 status interaction.**

| Effect | P-value |
| --- | --- |
| Ozone concentration | 0.1515 |
| 4 h vs. 22 h change | 0.0391 |
| Site | <.0001 |
| GSTM1 status | 0.2151 |
| Ozone concentration by GSTM1 status | 0.5995 |

GSTM1, glutathione S-transferase Mu 1 gene.

**Table Jjjj. Mixed model for change in platelet count (1000/uL) including ozone concentration by GSTM1 status interaction.**

| Effect | Estimate | Lower  95% CI | Upper  95% CI | P-value |
| --- | --- | --- | --- | --- |
| Intercept | -13.4 | -18.3 | -8.5 | <.0001 |
| 4 h change | 2.6 | 0.1 | 5.1 | 0.0391 |
| 22 h change | 0 | --- | --- | --- |
| Ozone exposure |  |  |  |  |
| 120 ppb | 0.3 | -3.7 | 4.4 | 0.8803 |
| 70 ppb | -1.2 | -5.2 | 2.8 | 0.5493 |
| 0 ppb | 0 | --- | --- | --- |
| Site |  |  |  |  |
| URMC | 5.4 | 0.2 | 10.6 | 0.0422 |
| UNC | 13.8 | 8.4 | 19.1 | <.0001 |
| UCSF | 0 | --- | --- | --- |
| GSTM1 status |  |  |  |  |
| Sufficient | -3.3 | -8.9 | 2.4 | 0.2507 |
| Null | 0 | --- | --- | --- |
| Ozone exposure by GSTM1 status |  |  |  |  |
| 120 ppb by sufficient | 2.3 | -3.8 | 8.5 | 0.4544 |
| 70 ppb by sufficient | -0.6 | -6.8 | 5.5 | 0.8369 |
| 0 ppb by sufficient | 0 | --- | --- | --- |
| 120 ppb by null | 0 | --- | --- | --- |
| 70 ppb by null | 0 | --- | --- | --- |
| 0 ppb by null | 0 | --- | --- | --- |

GSTM1, glutathione S-transferase Mu 1 gene; URMC, University of Rochester Medical Center; UNC, University of North Carolina; UCSF, University of California San Francisco.

**Table Kkkk. Type III sum of squares for change in vWF (ng/mL) including ozone concentration by age interaction.**

| Effect | P-value |
| --- | --- |
| Ozone concentration | 0.8486 |
| 4 h vs. 22 h change | 0.9652 |
| Site | 0.3598 |
| Age | 0.8370 |
| Ozone concentration by age | 0.0184 |

vWF, von Willebrand factor.

**Table Llll. Mixed model for change in vWF (ng/mL) including ozone concentration by age interaction.**

| Effect | Estimate | Lower  95% CI | Upper  95% CI | P-value |
| --- | --- | --- | --- | --- |
| Intercept | -166.7 | -5671.0 | 5337.7 | 0.9521 |
| 4 h change | -93.0 | -4313.1 | 4127.1 | 0.9652 |
| 22 h change | 0 | --- | --- | --- |
| Ozone exposure |  |  |  |  |
| 120 ppb | -1235.9 | -6394.7 | 3922.8 | 0.6368 |
| 70 ppb | 113.4 | -5018.8 | 5245.6 | 0.9653 |
| 0 ppb | 0 | --- | --- | --- |
| Site |  |  |  |  |
| URMC | -1026.6 | -6614.2 | 4561.0 | 0.7157 |
| UNC | 2879.6 | -2865.8 | 8625.1 | 0.3217 |
| UCSF | 0 | --- | --- | --- |
| Age | 495.1 | -340.8 | 1330.9 | 0.2421 |
| Ozone concentration by age |  |  |  |  |
| 120 ppb by age | -1381.7 | -2511.5 | -251.8 | 0.0167 |
| 70 ppb by age | 54.6 | -1072.7 | 1182.0 | 0.9242 |
| 0 ppb by age | 0 | --- | --- | --- |

vWF, von Willebrand factor; URMC, University of Rochester Medical Center; UNC, University of North Carolina; UCSF, University of California San Francisco.

**Table Mmmm. Type III sum of squares for change in vWF (ng/mL) including ozone concentration by sex interaction.**

| Effect | P-value |
| --- | --- |
| Ozone concentration | 0.7904 |
| 4 h vs. 22 h change | 0.9476 |
| Site | 0.4014 |
| Sex | 0.3958 |
| Ozone concentration by sex | 0.8219 |

vWF, von Willebrand factor.

**Table Nnnn. Mixed model for change in vWF (ng/mL) including ozone concentration by sex interaction.**

| Effect | Estimate | Lower  95% CI | Upper  95% CI | P-value |
| --- | --- | --- | --- | --- |
| Intercept | -2137.1 | -9030.4 | 4756.2 | 0.5392 |
| 4 h change | -141.3 | -4400.7 | 4118.1 | 0.9476 |
| 22 h change | 0 | --- | --- | --- |
| Ozone exposure |  |  |  |  |
| 120 ppb | 127.6 | -7888.6 | 8143.8 | 0.9750 |
| 70 ppb | 1935.0 | -6142.9 | 10013.0 | 0.6368 |
| 0 ppb | 0 | --- | --- | --- |
| Site |  |  |  |  |
| URMC | -1253.2 | -6834.3 | 4327.8 | 0.6563 |
| UNC | 2456.8 | -3341.5 | 8255.1 | 0.4018 |
| UCSF | 0 | --- | --- | --- |
| Sex |  |  |  |  |
| Female | 3936.8 | -3766.0 | 11640.0 | 0.3123 |
| Male | 0 | --- | --- | --- |
| Ozone concentration by sex |  |  |  |  |
| 120 ppb by female | -2851.3 | -13387.0 | 7684.4 | 0.5938 |
| 70 ppb by female | -2932.3 | -13447.0 | 7582.4 | 0.5826 |
| 0 ppb by female | 0 | --- | --- | --- |
| 120 ppb by male | 0 | --- | --- | --- |
| 70 ppb by male | 0 | --- | --- | --- |
| 0 ppb by male | 0 | --- | --- | --- |

vWF, von Willebrand factor; URMC, University of Rochester Medical Center; UNC, University of North Carolina; UCSF, University of California San Francisco.

**Table Oooo. Type III sum of squares for change in (vWF) ng/mL including ozone concentration by GSTM1 status interaction.**

| Effect | P-value |
| --- | --- |
| Ozone concentration | 0.7844 |
| 4 h vs. 22 h change | 0.9485 |
| Site | 0.3684 |
| GSTM1 status | 0.8161 |
| Ozone concentration by GSTM1 status | 0.5072 |

vWF, von Willebrand factor; GSTM1, glutathione S-transferase Mu 1 gene.

**Table Pppp. Mixed model for change in vWF (ng/mL) including ozone concentration by GSTM1 status interaction.**

| Effect | Estimate | Lower  95% CI | Upper  95% CI | P-value |
| --- | --- | --- | --- | --- |
| Intercept | -1073.1 | -7345.3 | 5199.1 | 0.7345 |
| 4 h change | -138.7 | -4393.1 | 4115.7 | 0.9485 |
| 22 h change | 0 | --- | --- | --- |
| Ozone exposure |  |  |  |  |
| 120 ppb | -595.8 | -7384.7 | 6193.1 | 0.8626 |
| 70 ppb | 2810.2 | -3959.6 | 9580.1 | 0.4136 |
| 0 ppb | 0 | --- | --- | --- |
| Site |  |  |  |  |
| URMC | -862.7 | -6446.7 | 4721.4 | 0.7594 |
| UNC | 2937.4 | -2817.2 | 8691.9 | 0.3129 |
| UCSF | 0 | --- | --- | --- |
| GSTM1 status |  |  |  |  |
| Sufficient | 2242.4 | -5455.4 | 9940.2 | 0.5639 |
| Null | 0 | --- | --- | --- |
| Ozone concentration by GSTM1 status |  |  |  |  |
| 120 ppb by sufficient | -2249.8 | -12798.0 | 8298.1 | 0.6742 |
| 70 ppb by sufficient | -6113.0 | -16582.0 | 4355.6 | 0.2506 |
| 0 ppb by sufficient | 0 | --- | --- | --- |
| 120 ppb by null | 0 | --- | --- | --- |
| 70 ppb by null | 0 | --- | --- | --- |
| 0 ppb by null | 0 | --- | --- | --- |

vWF, von Willebrand factor; GSTM1, glutathione S-transferase Mu 1 gene; URMC, University of Rochester Medical Center; UNC, University of North Carolina; UCSF, University of California San Francisco.

**Table Qqqq. Type III sum of squares for change in fibrinogen (µg/mL) including ozone concentration by age interaction.**

| Effect | P-value |
| --- | --- |
| Ozone concentration | 0.0391 |
| 4 h vs. 22 h change | 0.2172 |
| Site | 0.5518 |
| Age | 0.1877 |
| Ozone concentration by age | 0.3022 |

**Table Rrrr. Mixed model for change in fibrinogen (µg/mL) including ozone concentration by age interaction.**

| Effect | Estimate | Lower  95% CI | | Upper  95% CI | P-value |
| --- | --- | --- | --- | --- | --- |
| Intercept | -144.5 | -562.4 | 273.3 | | 0.4934 |
| 4 h post change | -197.0 | -512.2 | 118.1 | | 0.2172 |
| 22 h change | 0 | --- | --- | | --- |
| Ozone exposure |  |  |  | |  |
| 120 ppb | 328.7 | -56.6 | 714.0 | | 0.0940 |
| 70 ppb | -162.8 | -546.1 | 220.5 | | 0.4029 |
| 0 ppb | 0 | --- | --- | | --- |
| Site |  |  |  | |  |
| URMC | 151.5 | -278.0 | 580.9 | | 0.4850 |
| UNC | 240.3 | -201.2 | 681.8 | | 0.2821 |
| UCSF | 0 | --- | --- | | --- |
| Age | 51.9 | -11.2 | 115.0 | | 0.1059 |
| Ozone exposure by age |  |  |  | |  |
| 120 ppb by age | -63.3 | -147.6 | 21.1 | | 0.1414 |
| 70 ppb by age | -14.1 | -98.3 | 70.1 | | 0.7414 |
| 0 ppb by age | 0 | --- | --- | | --- |

URMC, University of Rochester Medical Center; UNC, University of North Carolina; UCSF, University of California San Francisco.

**Table Ssss. Type III sum of squares for change in fibrinogen (µg/mL) including ozone concentration by sex interaction.**

| Effect | P-value |
| --- | --- |
| Ozone concentration | 0.0537 |
| 4 h vs. 22 h change | 0.2182 |
| Site | 0.5717 |
| Sex | 0.9156 |
| Ozone concentration by sex | 0.9927 |

**Table Tttt. Mixed model for change in fibrinogen (µg/mL) including ozone concentration by sex interaction.**

| Effect | Estimate | Lower  95% CI | Upper  95% CI | P-value |
| --- | --- | --- | --- | --- |
| Intercept | -138.1 | -661.8 | 385.7 | 0.6014 |
| 4 h post change | -197.1 | -513.1 | 118.9 | 0.2182 |
| 22 h change | 0 | --- | --- | --- |
| Ozone exposure |  |  |  |  |
| 120 ppb | 292.5 | -302.2 | 887.2 | 0.3329 |
| 70 ppb | -181.2 | -780.5 | 418.2 | 0.5515 |
| 0 ppb | 0 | --- | --- | --- |
| Site |  |  |  |  |
| URMC | 179.1 | -256.8 | 615.0 | 0.4163 |
| UNC | 229.9 | -222.8 | 682.6 | 0.3154 |
| UCSF | 0 | --- | --- | --- |
| Sex |  |  |  |  |
| Female | -8.1 | -590.9 | 574.7 | 0.9781 |
| Male | 0 | --- | --- | --- |
| Ozone exposure by sex |  |  |  |  |
| 120 ppb by female | 42.5 | -739.3 | 824.4 | 0.9146 |
| 70 ppb by female | 40.3 | -740.0 | 820.5 | 0.9190 |
| 0 ppb by female | 0 | --- | --- | --- |
| 120 ppb by male | 0 | --- | --- | --- |
| 70 ppb by male | 0 | --- | --- | --- |
| 0 ppb by male | 0 | --- | --- | --- |

URMC, University of Rochester Medical Center; UNC, University of North Carolina; UCSF, University of California San Francisco.

**Table Uuuu. Type III sum of squares for change in fibrinogen (µg/mL) including ozone concentration by GSTM1 status interaction.**

| Effect | P-value |
| --- | --- |
| Ozone concentration | 0.0217 |
| 4 h vs. 22 h change | 0.2246 |
| Site | 0.3715 |
| GSTM1 status | 0.0061 |
| Ozone concentration by GSTM1 status | 0.1102 |

GSTM1, glutathione S-transferase Mu 1 gene.

**Table Vvvv. Mixed model for change in fibrinogen (µg/mL) including ozone concentration by GSTM1 status interaction.**

| Effect | Estimate | Lower  95% CI | Upper  95% CI | P-value |
| --- | --- | --- | --- | --- |
| Intercept | 54.6 | -408.9 | 518.2 | 0.8152 |
| 4 h change | -193.6 | -508.1 | 121.0 | 0.2246 |
| 22 h change | 0 | --- | --- | --- |
| Ozone exposure |  |  |  |  |
| 120 ppb | 95.1 | -406.8 | 597.1 | 0.7087 |
| 70 ppb | -35.8 | -536.3 | 464.8 | 0.8880 |
| 0 ppb | 0 | --- | --- | --- |
| Site |  |  |  |  |
| URMC | 247.4 | -165.1 | 659.9 | 0.2363 |
| UNC | 273.2 | -151.9 | 698.2 | 0.2048 |
| UCSF | 0 | --- | --- | --- |
| GSTM1 status |  |  |  |  |
| Sufficient | -575.3 | -1144.2 | -6.3 | 0.0476 |
| Null | 0 | --- | --- | --- |
| Ozone concentration by GSTM1 status |  |  |  |  |
| 120 ppb by sufficient | 542.4 | -237.5 | 1322.3 | 0.1715 |
| 70 ppb by sufficient | -274.1 | -1048.1 | 500.0 | 0.4854 |
| 0 ppb by sufficient | 0 | --- | --- | --- |
| 120 ppb by null | 0 | --- | --- | --- |
| 70 ppb by null | 0 | --- | --- | --- |
| 0 ppb by null | 0 | --- | --- | --- |

GSTM1, glutathione S-transferase Mu 1 gene; URMC, University of Rochester Medical Center; UNC, University of North Carolina; UCSF, University of California San Francisco.
