# Supplementary material for: Ozone effects on blood biomarkers of systemic inflammation, oxidative stress, endothelial function, and thrombosis: The Multicenter Ozone Study in oldEr Subjects (MOSES)
Source: PLoS One. 2019 Sep 25;14(9):e0222601. doi: 10.1371/journal.pone.0222601 (PMC6760801; doi:10.1371/journal.pone.0222601)
Supplement: S2 Checklist — (DOCX) [file pone.0222601.s003.docx]

CONSORT Checklist Notes

(a) The title does not specifically identify the study as a randomized trial, but that information is provided in the Methods.

(b) The study protocol, included with this submission, provides the minor changes made prior to and during subject recruitment.

(c) The sample size was determined using power calculations during study design, and an interim power calculation was also performed. These are detailed elsewhere and referenced in the manuscript (reference 36). This can be added to the manuscript if desired by the editor.

(d) Randomization in this study involved only the order of the three exposures for each subject. Further details about the randomization method can be added to the manuscript if desired by the editor.

(e) The source of funding for this study (Health Effects Institute) has been identified in the submission process and cover letter. Additional information can be added to the manuscript if desired by the editor.
