# Supplementary material for: Ozone effects on blood biomarkers of systemic inflammation, oxidative stress, endothelial function, and thrombosis: The Multicenter Ozone Study in oldEr Subjects (MOSES)
Source: PLoS One. 2019 Sep 25;14(9):e0222601. doi: 10.1371/journal.pone.0222601 (PMC6760801; doi:10.1371/journal.pone.0222601)
Supplement: S1 Protocol — (PDF) [file pone.0222601.s004.pdf]

ADDITIONAL MATERIALS 1

Section 1.1. MOSES Common Protocol and Changes to Protocol

Common Protocol for Multicenter Ozone Study in Elderly Subjects (MOSES)

This multicenter study will be conducted at three clinical centers/laboratories at the : University of Rochester Medical Center (UMRC), Rochester, NY; the University of California (UCSF), San Francisco, CA ; and the University of North Carolina (UNC) at Chapel Hill, NC.

1. RATIONALE FOR THE STUDY

It is well established that air pollution contributes to cardiovascular morbidity and mortality. Moreover, the American Heart Association recognizes that air pollution is a significant risk factor for cardiovascular disease. Particulate matter (PM) has been the major focus of previous acute air pollution studies. Less attention has been paid to acute cardiovascular responses to ozone (O_3), at least in part due to the notion that O_3 causes primarily local effects on the respiratory tract. However, several recent epidemiological studies reported increased risk of mortality associated with ambient exposure to O_3.

The maximum level of ozone in ambient air is regulated by the U.S. Environmental Protection Agency (EPA) as part of its mandate to set a National Ambient Air Quality Standard (NAAQS) for pollutants considered to be harmful to public health and the environment. The ozone NAAQS has been established largely based on data from human clinical studies showing decrements in lung function at ozone exposure concentrations as low as 0.07 ppm for 6.6 hours with extensive exercise. In March 2008 the EPA lowered the 8-hour ozone NAAQS to 0.075 ppm (from 0.080 ppm). More recently the EPA administrator proposed a revision to the 8-hour standard, within the range of 0.060-0.070 ppm, based on these lung function effects. However, it is unknown whether there are acute cardiovascular effects at these same ozone exposure levels. General pathways by which air pollutants, including ozone could cause vascular dysfunction include a) the induction of systemic inflammation and/or oxidative stress and b) alterations in autonomic balance. These initial responses could lead ultimately to endothelial dysfunction, impaired arterial dilation, cardiac arrhythmias, and increased pro-thrombotic activity in circulating blood, which could present a risk to individuals with atherosclerotic arterial disease.

The epidemiologic associations between exposure to ozone and increased risk of cardiovascular disease have raised new concerns about potentially harmful cardiovascular effects of this pollutant and there is a need for additional data from controlled human clinical studies, without many of the limitations common in observational studies, to assess potential ozone-mediated cardiovascular health effects. While controlled human exposure data on acute cardiovascular responses to ambient PM exposure have been accumulating due to directed research funding to support the epidemiological evidence of a PM-mortality association, the available data from controlled human exposures regarding the cardiovascular effects of O_3 are extremely limited. This study will address this data gap by investigating whether short-term controlled exposure to ambient levels of O_3 causes acute cardiovascular responses as assessed by changes in blood pressure, cardiac function, and systemic biomarkers of inflammation, endothelial dysfunction, and thrombosis.

The protocol for this study is based on the following suppositions:

- Although spirometric responses to ozone are known to be attenuated in older individuals, the elderly may be more susceptible to inflammatory and systemic vascular effects of ozone exposure compared with younger people.
- Detectable effects at low levels of inhaled ozone are more likely with exercise (increased ventilation) during the exposure.
- Subjects with pre-existing CV disease should be excluded for safety and to minimize variability

The study will be conducted in three research centers using a common protocol and common standard operating procedures. The subjects to be studied are healthy non-smoking adults aged 55 to 70 years who meet strict criteria for inclusion. They will be exposed in a chamber to clean air and two concentrations of ozone (70 and 120 ppb) for 3 hours while exercising intermittently. The lower concentration was chosen because it is between the current ozone NAAQS and the new proposed NAAQS. The higher concentration is within the range of concentrations many people are still exposed to in the United States (http://www.epa.gov/airquality/greenbook/oindex.html). To carry out the study each center's team consists of a multi-disciplinary group with experience in pulmonary and cardiovascular medicine, occupational/environmental medicine, cardiology and controlled human exposure studies of ozone health effects. A Data Coordination and Analysis Center will be responsible for assembling all the data from the centers and conducting the statistical analyses of the combined data set.

2. HYPOTHESES

Primary hypotheses

We hypothesize that ozone reaction products in airways surface liquid interact with lung cells (e.g., epithelial cells, macrophages, nociceptive sensory nerve endings, endothelial cells), and that these cells produce mediators that enter the pulmonary and systemic vasculature. In addition to its (local) effects on airway inflammation and lung function, ozone may have the following cardiovascular effects:

(1) altered autonomic balance (measured as changes in heart rate and heart rate variability (HRV)), cardiac arrhythmia, and repolarization

(2) altered systemic vascular function [measured as brachial artery flow-mediated dilation (FMD)~~without and with nitroglycerin (NTG)~~ – primary endpoint].

(3) pro-thrombotic vascular state (measured as increase in von Willebrand factor antigen in blood – primary endpoint). Secondary endpoints are: increase in microparticle-associated Tissues Factor activity and platelet activation.

Secondary hypotheses

We further hypothesize that:

- Markers of systemic oxidative stress and inflammation are correlated with the cardiovascular effects and degree of airway injury (measured as CC16 in blood) and airway inflammatory effects (neutrophils and cytokines in induced sputum).
- Cardiovascular effects are correlated with airway inflammatory effects.

Exploratory hypothesis

Subjects with GSTM1 null genotype (with impaired antioxidant defenses) will be more susceptible to effects of ozone.

3. CRITERIA FOR INCLUSION AND EXCLUSION

Inclusion

- ≥55 and ≤70 years of age; nonsmoking males and females of all ethnic backgrounds.
- Normal spirometry (FEV_1 and FVC ≥75% of predicted and FEV_1/FVC ≥0.65). Predicted values will be determined using the correction factor specific for African Americans and Latinos, but not for Asians, based on the NHANES III guidelines.
- Ability to complete the training exercise regimen chosen to induce an inspired ventilation rate of 13.6 to 15.5 L/min/m^2 body surface area (BSA) ATP (Ambient Temperature, Pressure) which is equivalent to 15 to 17 L/min/m^2 BTPS (Body Temperature, Pressure saturated with water vapor) without exceeding 80% of predicted maximal heart rate.
- Normal baseline 12-lead resting ECG, and absence of significant ST depression while performing the 15-minute required level of exercise targeted for the exposure period.
- Subjects must be able to avoid the medications supplements listed in Table 1 of section #7 for 1 week before the exposure.

Exclusion

- Non-English speaking.
- Including, but not limited to as ascertained by the physicians: Subjects with chronic cardiovascular (such as ischemic heart disease) or respiratory (such as asthma or COPD) disease; diabetes, or other organ or system dysfunction; cerebrovascular disease; active psychiatric disorders that would interfere with the subject's ability to understand and participate in the study. Subjects who have tested positive for a disease that affects the immune system (such as HIV, lymphoma, leukemia) or current drug or alcohol abuse (defined as having more than 3 drinks per day or being unable to abstain from alcohol for 3 days). Refer to the Initial Phone Screen Questionnaire for a detailed list.
- Subjects with atopy or allergic rhinitis will not be excluded as long as they do not require regular treatment with antihistamines or systemic steroids.
- Ever-smokers (smoked tobacco or marijuana during the last five years, or with history of >10 pack year for tobacco or > 1 joint year for marijuana, or living with a smoker who smokes inside the house).
- Subject having plasma cotinine level > 3ng/mL.
- BMI >35 and <18 (35 is the official cut off for class 1 obesity).
- Hypertension (defined as blood pressure >140 systolic or >90diastolic) or on anti-hypertension medications other than diuretics.
- Pregnancy or nursing (breastfeeding).
- On the following medications: prednisone, statins, beta-blockers, anticoagulants, current systemic estrogen therapy, tamoxifen. Subjects will not be asked to discontinue needed prescription medications for the purpose of this study. If any of these medications becomes necessary during the course of the study, the subjects will be excluded. Use of other medications will be considered on an individual basis (see section # 7).

- Subjects taking aspirin or PDE5 inhibitors must be willing to abstain from these medications during the week preceding each exposure (see section #7).
- Occupational exposures (exposed to high levels of vapors, dust, gases, or fumes on an on-going basis)

4. SUBJECT RECRUITMENT, INFORMED CONSENT, AND CONFIDENTIALITY
Subjects will be recruited by advertisement on local bulletin boards, by advertisement in local newspapers, in publications oriented to the required age group, and on Craigslist, notices placed in retirement complexes, and by recruitment from clinics at the investigator's institutions. Word-of-mouth contacts by previous subjects will be solicited. The initial screening will be done by phone.

Number of subjects in each center: n=30 (who have completed all three exposure sessions). Approximately 140 subjects in total would be recruited to achieve the target number of subjects completing the three exposure sessions.

Informed Consent
During the screening visit 1 (see below), the investigator will obtain all informed consents from the study subject by means of a dated and signed consent approved by the local IRB/EC. The study subject will be given a copy of all signed informed consent forms. The informed consent process will be performed in accordance with the International Conference on Harmonization guidelines for Good Clinical Practice (GCP) and federal laws and regulations.

Subject confidentiality
Patient confidentiality will be maintained according to ICH guidelines for GCP and applicable local and national data protection laws. A study identification number will be assigned to each subject. The link between patient identifiable information and I.D. number will be stored only at the clinical center where the subject receives his/her care, thereby ensuring that all data transferred from a subject's medical records to a study report form and any process derived from the study report form is handled confidentially.

5. SCREENING AND TRAINING VISITS
Subjects who volunteer to participate will visit the laboratory or clinical research center (CRC) for screening and training on two separate days.

Screening visit – Visit #1 (baseline subject characterization and questionnaires)
A *Health and Home Screening Questionnaire*, and a physical examination, will be administered to obtain the following: medical history, cardiovascular and respiratory health status, occupational history, history of allergies, use of medications and vitamin supplements, diet, alcohol use, occupational exposure, SES, home characteristics/heating type and air conditioning use. Particular attention will be given to medical history or physical findings suggesting arterial vascular or cardiac disease, or unexplained syncope or unusual dyspnea as well as other risk factors for cardio-vascular disease. Height, weight, and vital signs will be measured. In a woman who has not yet completed menopause, a urine pregnancy test will be done.

A baseline 12-lead ECG will be taken at rest. Trained personnel will be responsible for attaching the leads. If the instrument interpretation is normal, the ECG will be reviewed and signed by the study

physician. If any abnormalities are detected, it will be reviewed by the cardiologist. In consultation with the cardiologist, the local study physician will make the decision whether the subject should be included.

Spirometry will be performed on a spirometer that meets American Thoracic Society performance criteria using NHANES III predicted values.

Venous blood will be collected for analyses of cotinine (markers of exposure to tobacco smoke), total and differential blood counts, metabolic profile, and lipid profile (cholesterol, HDL, and triglycerides).

Training visit – Visit #2
Vital signs will be measured. In a woman who has not yet completed menopause, a urine pregnancy test will be done. The subject will exercise (on a treadmill or a stationary bicycle) for 15 minutes, rest for 15 minutes, and exercise another 15 minutes. The level of exercise will be adjusted to achieve the target inspired minute ventilation (V_I) of 13.6 to 15.5 L/min/m^2 BSA (ATP) which is equivalent to target expired minute ventilation (V_E) of 15 to 17 L/min/m^2 BSA (BTPS) assuming the correction factor of 1.1 (the same that will be required during the exposure period), measured by a mouthpiece and pneumotachograph, while not exceeding 80% of the predicted maximum heart rate (HR). 80% of the predicted maximum heart rate per minute will be calculated from the following formula HRmax= 208 – 0.7 x age (yrs) (Tanaka et al, J Am Coll Cardiol 37:153, 2001). For example, a 70-year old subject would have a maximum predicted heart rate of 159 beats/min and the exercise required to achieve a minute ventilation of at least ~~15~~ 13.6 L/min/m^2 BSA (ATP) in that subject must not cause the heart rate to exceed 127 beats/min.

Blood pressure will be measured at rest pre- and post-exercise and possibly during exercise if sudden changes in HR are observed. 3-lead cardiac monitoring will be performed during the 45-minute exercise/rest/exercise period. A rhythm strip printout will be obtained before the exercise and every 5 minutes during the exercise. Trained personnel will be responsible for attaching the leads and a physician will be present for the first 15-minute exercise period, and in the building, reachable by pager, during the rest of the visit. The cardiac rate and rhythm will be monitored in real time by the study personnel and by the physician while present during the first exercise period. The monitor's printout will be reviewed and signed by the study physician; if the subject can also complete the level of exercise needed, the subject would be entered into the study. If any abnormalities such as ST depression, arrhythmias, or other abnormalities, are detected, they will trigger a review by the cardiologist. In consultation with the cardiologist, the local study physician will make the decision whether the subject should be included. *An Ozone Monitor Activity Diary* and a personal exposure sampler (PES) will be given to the subject with instructions for filling the diary and wearing the PES ~~it~~. The sampler should be worn for the 3 days preceding the pre-exposure day (starting at noon).

6. EXPOSURE VISITS AND TIMELINE OF MEASUREMENTS
Subjects will be exposed to clean air, 70 ppb (low), and 120 ppb (high) ozone for 3 hours in a randomized order. Each exposure session involves 3 consecutive days as shown below. There will be a minimum 2-week period between the exposure sessions.

Exposure (order will be randomized)	Pre-exposure day	Exposure day	Post-exposure day
Clean Air	Visit 3	Visit 4	Visit 5
70 ppb ozone	Visit 6	Visit 7	Visit 8

120 ppb ozone	Visit 9	Visit 10	Visit 11

Prior to visit 3, 6, and 9 the subjects will receive reminder phone calls and will be asked to answer the *Before Pre-exposure Visit Phone Questionnaire*, which includes questions about the subject's health and medication use and gives a few reminders about the upcoming visit or, if necessary, provides the opportunity to reschedule the visit.

Visit 3, 6, and 9 - pre-exposure day. The subject will arrive at the laboratory or CRC at 11:30 am –12:00 noon. A low-fat lunch (25-30% fat) will be provided. The *Ozone Monitor Activity Diary* and PES will be collected and the subject will answer *Pre-Exposure Day Health Questionnaire*. In a woman who has not yet completed menopause, a urine pregnancy test will be done.

The following procedures will be carried out: measurement of vital signs, venous blood draw (up to 30 mL), ultrasound measurement of flow-mediated (FMD) ~~and nitroglycerin-mediated (NTG)~~ brachial artery dilation ultrasound (BAU). A box dinner will be provided. The subject will spend the night at the CRC or at a nearby hotel. Before discharge the subjects will be reminded that the researchers believe it is important to avoid any environmental exposure that might possibly affect his/her reaction to the controlled exposure scheduled for the following morning. For that reason they are providing a boxed supper and close-by sleeping accommodation in a nonsmoking environment. They will stress that the subject's thoughtfulness and cooperation is very much appreciated.

Visit 4, 7, and 10 – exposure day. The subject will arrive at the laboratory or CRC at 7:00 – 7:30 am. A regular breakfast will be provided. The following events will occur: blood pressure (and other vital signs) will be measured; the subject will complete the symptom questionnaire; the subject will wear a Holter monitor, heart rate variability (HRV) will be measured (the subject lies quietly for 5 minutes then the ECG is recorded for 5 minutes under tidal breathing) and continuous ECG recording will begin; spirometry will be measured~~.~~

The exposure will start between 8:00 and 8:30 am and will last 3 hours. The subject will alternate a 15-minute exercise period with a 15-minute rest period. Subjects will be blinded to ozone dose and order of exposure. Assignment of each subject to the exposures will be randomized (see Section #15).

Minute ventilation will be measured with pneumotachograph between 8 and 10 minutes of the first exercise period and during the last 2 minutes of the first, second, fourth, and sixth exercise period and the exercise load adjusted as needed. Blood pressure will be measured during a rest period, 5 minutes before the first, third and fifth exercise period. Heart rate will be measured twice during the first exercise period and once during each of the other exercise periods.

The symptom questionnaire will be filled out by the subject in the chamber during the last 15 minutes of exposure. Immediately after exposure blood pressure (and other vital signs) will be measured, then HRV and spirometry. A low-fat (25-30% fat) lunch will be provided.

Approximately 3.5 hours after the end of the exposure the sequence of the measurement will be: HRV, venous blood draw (up to 30 mL), BAU FMD ~~and NTG-mediated dilation~~), symptom questionnaire, ~~and~~ blood pressure ~~(~~and vital signs~~)~~. The subject will be sent home wearing the Holter monitor. The subject will leave the laboratory or CRC at around 4:00 pm.

Visit 5, 8, and 11 –post-exposure day. The subject will arrive at the laboratory or CRC at 8:00 am. The subject will need to be fasting from 7:30 am 2 hours prior to sputum induction, in order to avoid contamination of the sputum specimen. The sequence of measurements will be: vital signs, symptom questionnaire, HRV, venous blood draw (up to 30 mL), and spirometry/sputum. Blood pressure may be measured. The subject will leave the laboratory and CRC at around 10:30 am and will be given the *Ozone Monitor Activity Diary* and PES (and associated instructions) to wear before the next exposure visit (with exception of visit 11).

7. ACTIVITY, DIET, AND OTHER REQUIREMENTS ON DAY(S) PRIOR TO EXPOSURE

Subjects should abstain from caffeinated beverages (i.e., coffee, tea, energy drinks, and sodas) and alcoholic beverages starting with lunch the day before the exposure through the post-exposure day. Subjects must be able to avoid the medications and supplements listed in Table 1 below for 1 week prior to the exposure. In addition, subjects will not be studied within 6 weeks of a respiratory infection. Use of diuretics to control mild hypertension will be permitted.

Subjects will wear the PES for the 3 days, starting at noon, before the pre-exposure day. Subjects will be asked to abstain from exercising on the day before the exposure. They will fill out the *Ozone Monitor Activity Diary* while wearing PES.

Table 1

For one WEEK before each exposure day
Non-steroidal anti-inflammatory drugs such as ibuprofen, naproxen, aspirin
Phosphodiesterase 5 (PED5) inhibitors such as Viagra, Cialis, Levitra
Supplemental vitamins
Antihistamines
Anti-oxidants
Fish oil
Niacin
Arginine
Over-the counter decongestants

8. CARDIOVASCULAR AND PULMONARY OUTCOMES

The majority of the endpoints will be measured on the day before the exposure and at 4 and 22 hours after the exposure. Markers of inflammation in sputum will be measured only once because sputum will be induced only at 22 hours after the exposure.

Health symptoms. These will be assessed using a symptom questionnaire.

Endothelial and vascular function

- Blood pressure.
- Brachial artery flow-mediated dilation (FMD) measured by ultrasound (BAU)

- ~~Brachial artery nitroglycerine (NTG)-mediated dilation measured by ultrasound (BAU-NTG)~~
- Brachial artery blood flow with Doppler probe immediately prior to the artery dilation measurements.

Cardiac function. Will be measured by electrocardiogram (24 hour Holter with 12-lead recording)

- Heart rate and HRV parameters in both the time domain (SDNN, PNN50, RMSSD) and the frequency domain (VLF, LF, HF, TP, LF/HF).
- Repolarization changes
- Arrhythmia
- ST-segment changes

Blood markers. The volume of venous blood to be collected will be a maximum of 150 ml over 3 days and no more than 500 mL over the study.

<u>Endothelial function markers</u>

- Microparticle production. Microparticle production by the vascular endothelium is a measure of endothelial cell activation and/or injury .
- Endothelin-1 and P-selectin. These molecules mediate various aspects of endothelial function.

<u>Prothrombotic vascular state</u>

- Von Willebrand factor (vWF) antigen- primary endpoint. vWF is a blood glycoprotein that plays a critical role critical in the initial stages of blood clotting. Its level in plasma (vWF antigen) will be measured in a commercial laboratory.
- Fibrinogen. It is a coagulation factor and an acute phase reactant and contributes to risk of cardiovascular outcomes.
- Tissue factor (TF) activity. Tissue factor is a potent pro-coagulant. The activity of TF associated with microparticles will be measured at UNC.
- Platelet activation. The activation of platelets is a critical component of atherothrombosis, and could be increased by air pollutants, including ozone.

<u>Oxidative stress and systemic inflammation markers</u>

- Several soluble markers will be measured [e.g., 8-isoprostane, nitrotyrosine, C-reactive protein (CRP), and interleukin-6 (IL-6)].
- Differential blood cell counts.

<u>Lung injury markers</u>

- Serum Clara Cell protein 16 (CC16). CC16 is a marker of the integrity of the air-blood barrier that is known to be affected by ozone.

Markers of airway inflammation (in sputum samples)

- Differential white cell counts. Total protein, IL-6, IL-8, TNF-α, CD40 ligand. These are well-documented markers of airway inflammation.

Lung function (spirometry)

- FVC, FEV_1, $FEV_{25/75}$. Spirometry will be performed on an ATS-approved spirometer using modified ATS performance criteria and NHANES III predicted values.

Marker of genetic susceptibility to ozone

- GSTM1 genotype in stored blood samples. *GSTM1* is a gene whose product, glutathione S-transferase, is an antioxidant enzyme that may be involved in susceptibility to ozone. The null polymorphism of this gene is very common in human populations but does not have any known clinical significance.

9. SAMPLES or DATA TO BE ANALYZED IN A CORE LABORATORY

- BAU-FMD ~~and BAU-NTG~~ (UCSF)
- Holter ECG (URMC)
- Blood markers: Endothelin-1 and P-selectin, vWF factor (level), fibrinogen, 8-isoprostane, nitrotyrosine, CRP, IL-6 (clinical lab)
- Blood markers: microparticle-associated TF activity (UNC) (vWF multimers may be also measured)
- Sputum markers of inflammation: Differential cell counts (each center); total protein, IL-6, IL-8, TNF-α (UNC)
- PES (Research Triangle Institute, RTP, NC)
- GSTM1 genotypying (clinical lab)

10. TISSUE BANKING

Blood and sputum samples will be banked for measurement of soluble markers of airway injury, inflammation and oxidative stress, and gene polymorphism for antioxidant enzymes.

11. ASSESSMENT OF EXPOSURE PRIOR TO THE CHAMBER EXPOSURE

Daily data on ambient air quality for ozone, $PM_{2.5}$, and PM_{10} will be obtained from local monitoring sites.

The subject will wear a PES sensitive to ozone and nitrogen dioxide for 3 days prior to the pre-exposure day. The rationale is that it is possible that the health measurements will be influenced by both indoor and outdoor sources of pollution prior to the exposure sessions, including environmental tobacco smoke, occupational exposures, and/or other indoor PM sources that will not be captured with the ambient PM monitoring data. Possible indoor exposures (such as kerosene heaters) will be identified through the activity diary associated with the PES measurements.

12. EXPOSURE GENERATION AND MONITORING SYSTEM

The air entering the exposure chamber will be purified by passing it through several filters (Purafil, charcoal, HEPA). Ozone will be generated by a high-voltage corona-spark, silent discharge ozonator. Temperature and relative humidity will be maintained at approximately 22 ^{o}C and 40%, respectively.

13. DATA MANAGEMENT, ORGANIZATIONAL STRUCTURE, AND DATA SAFETY MONITORING PLAN

A plan for data collection, transfer, and management will be developed in collaboration with the Data Coordinating and Analysis Center (DCAC) at the (New England Research Institute, Watertown, MA.)

The study will be overseen by the HEI Research Committee, a group of scientists with diverse expertise, which is responsible for developing and managing HEI research activities. Two special bodies of scientists will be set up to coordinate the conduct of the study, monitor the data collection, and discuss

issues related to subject safety and publication of the data: a Data Monitoring Board and a Steering Committee. The role of these two groups is summarized below.

The Data Monitoring Board (DMB) is composed of statisticians and cardiologists (including members of the HEI Research Committee and outside experts appointed by the Research Committee), the Data Coordinating and Analysis Center team, and HEI staff. The DMB will establish procedures for the timely submission of data from the centers, monitor the quality of the data, monitor the implementation of the data analysis plan, and monitor subject safety through review of data on adverse events. The DMB may also perform interim analyses of the data to assess whether early stopping of the study might be warranted. The DMB will report any significant findings directly to the HEI Research Committee and, as necessary, with the three clinical centers' investigators.

The Steering Committee is composed of the Principal Investigators of the four centers and members of the HEI staff and one member of the HEI Research Committee. The Steering Committee will implement the analytical plans, review reports to the individual institutional IRBs of adverse events and protocol violations, report adverse events and protocol violations to the DMB in a timely fashion, prepare and decide on authorship of reports and publications, and discuss and make decisions about requests for biological samples, data, and specific analyses (from either the individual centers' investigators or other researchers).

Data Safety Monitoring Plan

Adverse events will be graded according to the following scale:
Grade 1 Mild: transient mild discomfort; no limitation activity; no medical intervention or therapy required.
Grade 2 Moderate: mild to moderate limitation in activity, some assistance may be needed; minimal noninvasive intervention or therapy (such as over-the-counter medication, application of ice, etc.) is allowed.~~no medical intervention or therapy required.~~
Grade 3 Severe: marked limitation in activity; some assistance required; medical intervention or therapy required, hospitalization possible.
Grade 4 Life-threatening: extreme limitation in activity, significant assistance required; significant medical intervention or therapy required; hospitalization probable.

Subjects will be encouraged to contact us if any adverse effects are noted after visits to the laboratories. They will have a 24-hour contact and pager numbers to report any potential adverse events that occur after study visits. If any subject has more than mild adverse effects from participation in the protocol or if several subjects have mild adverse effects, we will consider changing the protocol to improve subject safety and comfort. All adverse events that occur during the course of the study will be reported to the relevant IRB in accordance with current institutional guidelines. Subjects with two or more Grade 3 adverse events or with one Grade 4 adverse event will be withdrawn from the study. Information on adverse events will also be collected on a standardized data form and submitted to the Advance Data Entry and Protocol Tracking (ADEPT) system (at the Data Coordinating and Analysis Center) within 5 working days of occurrence (or as soon as the center is notified of the occurrence), with the exception of grade 3 and 4 events, which will be reported within 24 hour of occurrence (or as soon as the center is notified of the occurrence). The Data Coordinating and Analysis Center will periodically generate a report of all adverse events and submit it to the Steering Committee, the DMB, and HEI. As soon as the occurrence of a grade 3 or 4 event is reported to the ADEPT system, the Steering Committee and HEI will

be notified via email and the aggregated Adverse Events report will be generated and become available for viewing.

Frequency of Safety Reviews: Safety monitoring data, consisting of reports of adverse effects, as well as of study enrollment and dropout data , provided by the Data Coordinating and Analysis Center will be reviewed on the regular (at least monthly) Steering Committee teleconferences and reported to the DMB on a semi-annual basis.

Stopping Rules: We will stop the entire study (at all participating centers) in case of the following adverse events:

1) Death directly or indirectly due to any of the procedures performed in this protocol: e.g., exercise, medications administered, etc. (any Grade 4 adverse events).
2) Hospitalization directly or indirectly due to any of the procedures performed in this protocol: e.g., exercise, medications administered, etc. (any grade 4 adverse events).
3) Five subjects with Grade 3 events (described above).

The study will not be resumed until all the adverse event information has been discussed with the Steering Committee, the DMB, and the relevant IRBs and HEI have concurred with resumption of the study.

14. RANDOMIZATION AND DATA ANALYSIS

There are six possible ways of ordering the receipt of three different levels of ozone. Each ordering defines an exposure plan. Participants will be randomly assigned to one of the six plans using permuted blocks with institutional balancing. This ensures that that within each center approximately the same number of subjects would receive each exposure plan. For randomization by permuted blocks, the participants are randomized in blocks of six so that within each block of six participants all exposure plans are used. The assignment of the exposure plans within a block of six participants is randomly determined.

The primary analysis for this multi-center crossover design is a mixed effects linear model. For each of the three exposures (no ozone, 70 ppb, 120 ppb), most outcome variables will be measured pre-exposure, 4 hours after exposure, and 22 hours after exposure. The 4 hr and 22 hr measures will be the dependent variables with the baseline measure as a covariate. That will allow us to assess the effect of ozone on change from baseline to the two post-exposure measures. We will assume an autoregressive correlation structure among the repeated factors. If ozone exposure does affect how much the outcome variable changes from pre-exposure to one or both post-exposure times, we would expect to find an interaction effect of time by ozone level to be statistically significant.

The linear model for this design is:

$$y_{i:jk(l)t} = \mu + y_{i:jk(l)0} + \tau_k + \gamma_{(l)} + \alpha_j + \beta_{i:j} + \theta_t + \tau\theta_{kt} + \varepsilon_{i:jk(l)t} ,$$

where $y_{i:jk(l)t}$ is the outcome measure for subject i in center j at ozone exposure k preceded by exposure l at time t. Specifically, $y_{i:jk(l)0}$ is the outcome pre-exposure level of the outcome measure (t=0). τ_k is the direct effect of treatment k and $\gamma_{(l)}$is the residual or carry-over effect of treatment l. α_j is the effect of center j and $\beta_{i:j}$ is the effect of subject i in center j, θ_t is the effect of time t and $\tau\theta_{kt}$ is the interaction effect of treatment k at time t.

If the outcome measure is Gaussian, the model will be estimated by a least squares linear model. If the outcome measure is continuous but not normally distributed, we may consider a normalizing transformation. If the outcome measure is dichotomous we will use a general linear model with binary errors and a logit link function. Using this basic model we can include additional terms including stratification or other effects (e.g. gender) and interaction effects for treatment with stratification factors or center. If the "wash-out" period is sufficiently long we may omit the residual effect from the model, although there is little or no loss in efficiency in including it even if it is 0.

The primary outcome analysis will be conducted on the 90 participants who complete all three exposure sessions. In order to achieve 90 participants with complete data, we will recruit approximately 140 (64% completion rate) men and women. Due to randomization, this attrition will not affect the internal validity of the study but it may affect the generalizability of the results. We will compare the characteristics of those who complete the protocol compared to those who do not using logistic regression analysis. Factors to be examined include baseline characteristics of the participants including age, gender and selected measures of cardiovascular health as well as ozone exposure at the initial session and study center.

15. SAFETY OF THE SUBJECT

The subjects to be included in the study will need to meet strict inclusion criteria. Individuals with history of cardiac or pulmonary diseases, current smoking, diabetes, asthma or obesity will be excluded. The subjects will not be asked to abstain from needed medications. Individuals unable to perform mild exercise (defined as a level of exercise that causes the subject to achieve a minute ventilation volume of 15-17 L/min/m^2 BSA) will also be excluded. In addition, during the screening visit an electrocardiogram will be obtained on subjects both at rest and during exercise to detect possible abnormalities (such as ST-segment depression or arrhythmia) in their cardiac function that may put them a risk if exposed to ozone. A cardiologist will review any abnormal ECGs. Trained personnel will be performing all the clinical procedures and a physician will be on call during the period the subject is at the laboratory or CRC.

As an additional safety precaution, the 24hr Holter ECG report generated by the ECG ~~HRV~~ core laboratory (URMC) will be reviewed by the core cardiologist and the results of this review and a report indicating whether it is safe to proceed with the next exposure will be provided to the center's clinical coordinator before the subject is allowed to continue with the next exposure. If clinically indicated, the subject will be referred to his/her physician.

16. PROTECTION AGAINST RISKS

The risks of breathing ozone for 3 hours with intermittent exercise should be minimal. Ozone can cause mild chest tightness, cough, and difficulty taking a deep breath in responders, but the relatively low-level exposures to be used in the proposed study are unlikely to cause these symptoms especially in older adults. The ambient background ozone concentration frequently reaches 70 ppb (our low ozone exposure level) during summer time. The higher concentration to be used in the study, 120 ppb, is frequently exceeded downwind of major metropolitan areas, such as Los Angeles, in the summer. Many clinical studies of ozone have been conducted in these and other laboratories, some at much higher concentrations than planned here; there have been no serious adverse events associated with these exposures. Increased levels of air pollution (especially PM) have been associated in population studies with increased risk for acute cardiac events in the elderly and people with underlying cardiovascular disease, but these proposed experimental exposures will not represent a significant risk in healthy subjects.

In the environmental chamber, monitoring facilities provide continuous measurements of pollutant concentrations. The exposure will be discontinued if significant symptoms occur, at the subject's request or by a decision of the PI. The exposure facility is located on the medical center campus. Physician availability is assured at all times, and appropriate treatment is available if required.

Study risks include: physical injury/discomfort, stress, discovery of an unknown condition, invasion of subject privacy, and questions about topics that may be sensitive or offensive. No information with any personal identifier will be released from the investigator without specific consent from the study participant. Confidentiality will be further protected by using an alphanumeric code, without the subject's name, to identify all data and biological samples. Access to subject identifiers will be restricted to the PI and study coordinator. All medical records and personal study data are available to each participant in the study or any physician they choose upon written request and signed consent by the subject. We will also obtain a Certificate of Confidentiality.

Occluding circulation to the forearm with a blood pressure cuff for 5 minutes during the BAU FMD measurement can cause numbness, tingling, and discomfort in the hand. This resolves after release of the cuff, without residual effects. ~~The test will be repeated following administration of nitroglycerin under the tongue (BAU-NTG). NTG is sometimes associated with a short-lasting headache, dizziness or flushing.~~ ~~After the measurement the subject will rest quietly for ~5 min so that the effects of the drug will have worn off before leaving the laboratory. We will emphasize to each subject the importance of avoiding the use of phosphodiesterase-5 (PDE5) inhibitors (e.g., Cialis, Viagra, Levitra, etc.) before arriving for the study, because of potential interactions of these drugs with nitroglycerin.~~

Phlebotomy can cause local pain or bruising, and rarely syncope.

Sputum induction can cause bronchoconstriction in people with asthma; subjects with asthma will be excluded from this study, and spirometry will be obtained immediately before sputum induction to screen for unexpected baseline bronchoconstriction. However, if an unexpected severe bronchoconstriction during or following induction occurs it will be treated with a bronchodilator.

17. RISKS IN RELATION TO POTENTIAL BENEFITS
We believe that by identifying mechanisms of pollutant-induced health effects, more appropriate standards, strategies, and treatments can be developed to protect the general public health. Furthermore, these studies will provide important insights into mechanisms involved in the cardiovascular effects of air pollution exposure. There are no anticipated benefits to the subjects. We believe the relatively minimal risks of these studies are balanced by the importance of the knowledge gained about air pollution health effects in general, and the potential to protect the most susceptible people from those effects.

18. RECRUITMENT OF VULNERABLE SUBJECTS
Students or employees under the direct supervision of the investigators may not participate in this study. Decisionally-impaired adult subjects or those with questionable capacity to consent will not be included in the study.

19. ALTERNATIVE TO PARTICIPATION
The alternative to participation in the study is not to participate.

20. COSTS TO SUBJECTS
There will be no costs to subjects to participate in this study.

LIST OF CHANGES TO COMMON PROTOCOL OF MARCH 23 2012 AND OTHER DOCUMENTS SUBMITTED TO IRBs

(March 2012 - January 2013)

October 11, 2012

Changes to documents for IRB amendment of October 2012

Common Protocol 23MAR2012. The second paragraph of section 15 has been modified to indicate that the cardiologist of the ECG core laboratory will provide a report indicating whether it is safe to proceed with the next exposure. As a result, the review of the Holter ECG report by the study cardiologist was deemed no longer necessary. The new file is Common Protocol 11OCT2012.

Another change is to clarify the unit of minute ventilation. The change indicates that the target MV of 15 to 17 L/min//m^2 BSA is in BTPS (Body Temperature, Pressure saturated with water vapor) units. MV acquired under ATP (Ambient Temperature, Pressure) conditions by some devices need to be converted to BTPS conditions. The protocol includes the conversion from ATP to BTPS.

Initial Phone Screen Questionnaire 18JUN2012. The following medication type was deleted from section B.12 of the questionnaire: Osteoporosis drugs. For neurologic drugs instructions were added to see the Medication List because some types of neurologic drugs are allowed. The new file is Initial Phone Screen Questionnaire 10OCT2012.

Medication List for Phone Screen 01FEB2012. New file is: Medication List for Phone Screen 10OCT2012-Vesion B). The following prescription medications were removed from the list of medications that would exclude a subject:

Alendronate
Alprazolam
Amoxicillin
Amoxicillin_Clav
Azithromycin
Bupropion_Hcl
Fluoxetine
Gabapentin
Hydrocodone
Lorazepam
Medroxyprogesterone
Omeprazole
Ranitidine
Zolpidem

December 21, 2013

Documents for IRB amendment of January 2013

- **Common Protocol 21DEC2012**. Deleted any reference to the NTG-mediated brachial artery dilation. The decision to stop the procedure was made in the conference call of December 19, 2012. Also the definition of Grade 2 adverse events has been changed as follows:

  MOSES Grade 2 Moderate: mild to moderate limitation in activity, some assistance may be needed; ~~*no medical intervention or therapy required.*~~ *minimal noninvasive intervention or therapy (such as over-the-counter medication, application of ice, etc.) is allowed.*

- **Informed Consent** (center specific). The description of the NTG procedure (pre- and post-exposure) should be deleted. A sentence should be added to explain that the FMD procedure may be repeated. The UCSF sonographer suggested adding the following text: "The procedure may need to be repeated if the pictures are not of good quality. You will rest for 20 minutes before starting the procedure again."

- **Adverse Event Report Form**. The definition of Grade 2 events needs to be changed for consistency with the protocol.

- **Initial Phone Screen Questionnaire 10DEC2012** (the shading in the Yes box for thyroid disease was removed. Physician should probe during physical exam)

December 21, 2013- Revised January 24, 2013

Documents for IRB amendment of January 2013 – NEW changes, January 24, 2013 (requested by UR IRB)

- **Common Protocol 24JAN2013**.
  - Exclusion criteria, second bullet. Added a reference to the Initial Phone Screen Questionnaire because it provides more details of the exclusionary criteria
  - Page 14, second paragraph. Deleted sentence the last two sentences because they contain a reference to NTG.

- **Initial Phone Screen Questionnaire 24JAN2013**
  - Page 4, C11, thyroid disease. Added "Stable treated hypothyroidism would not exclude subject"

Documents for IRB amendment of January 2013 – December 21, 2012

- **Common Protocol 21DEC2012**. Deleted any reference to the NTG-mediated brachial artery dilation. The decision to stop the procedure was made in the conference call of December 19, 2012. Also the definition of Grade 2 adverse events has been changed as follows:

  MOSES Grade 2 Moderate: mild to moderate limitation in activity, some assistance may be needed; ~~no medical intervention or therapy required.~~ minimal noninvasive intervention or therapy (such as over-the-counter medication, application of ice, etc.) is allowed.

- **Informed Consent** (center specific). The description of the NTG procedure (pre- and post-exposure) should be deleted. A sentence should be added to explain that the FMD procedure may be repeated. The UCSF sonographer suggested adding the following text: "The procedure may need to be repeated if the pictures are not of good quality. You will rest for 20 minutes before starting the procedure again."

- **Adverse Event Report Form**. The definition of Grade 2 events needs to be changed for consistency with the protocol.

- **Initial Phone Screen Questionnaire 10DEC2012** (the shading in the Yes box for thyroid disease was removed. Physician should probe during physical exam)
